# Dual Responsive Polymersomes for Gold Nanorod and Doxorubicin Encapsulation: Nanomaterials with Potential Use as Smart Drug Delivery Systems

**DOI:** 10.3390/polym11060939

**Published:** 2019-05-30

**Authors:** Melissa DiazDuarte-Rodriguez, Norma A. Cortez-Lemus, Angel Licea-Claverie, Jacob Licea-Rodriguez, Eugenio R. Méndez

**Affiliations:** 1 Centro de Graduados e Investigación en Química. Tecnológico Nacional de México/Instituto Tecnológico de Tijuana, A.P. 1166, C.P. 22000 Tijuana, B. C., México; melissa.duarte@tectijuana.edu.mx (M.D.-R.); ncortez@tectijuana.mx (N.A.C.-L.); 2 División de Física Aplicada. Centro de Investigación Científica y de Educación Superior de Ensenada, Carr. Ensenada-Tijuana No. 3918, C.P. 22860 Ensenada, B. C., México; jlicea@cicese.mx (J.L.-R.); emendez@cicese.mx (E.R.M.); 3 Cátedras Conacyt, Centro de Investigación Científica y de Educación Superior de Ensenada, 22860 Ensenada, B. C., México

**Keywords:** Block copolymers, stimuli sensitive polymers, gold nanorods, photothermal therapy, drug delivery systems, polymersomes

## Abstract

In the present study, poly(ethylene glycol)-*b*-poly(*N,N*-diethylaminoethyl methacrylate) (PEG-*b*-PDEAEM) amphiphilic block copolymers were synthetized by reversible addition-fragmentation chain transfer (RAFT) polymerization using two different macro chain transfer agents containing PEG of 2000 and 5000 g/mol and varying the length of the PDEAEM segment. From the obtained block copolymers, polymersome type nanometric aggregates were obtained by two different techniques. By direct dispersion, particle diameters around 200 nm were obtained, while by solvent exchange using THF and water, the obtained diameters were around 100 nm. These block copolymers were used to encapsulate gold nanorods and doxorubicin (DOX) with good efficiencies to obtain nanomaterials with potential use as dual stimuli-sensitive drug delivery systems for combined anticancer therapies. Drug delivery studies showed that the release rate of DOX was accelerated when the pH was lowered from 7.4 to 5.8 and also when the systems were irradiated with a NIR laser at pH 7.4. The combination of lower pH and near infrared (NIR) irradiation resulted in higher drug release only in the case of polymersomes with lower molecular weight PEG.

## 1. Introduction

In aqueous environments, amphiphilic block polymers have the ability to self-assemble into a variety of polymeric aggregates; the most common cases are micelles, where the hydrophobic blocks of the copolymer form the core while the hydrophilic blocks form the outer shell [1,2,3]. Larger, but still nanometric aggregates, such as polymersomes, are self-assembled hollow vesicles surrounded by amphiphilic block copolymer membranes that can be formed by bilayers or complex interdigitated structures [4]. Currently, there is a great deal of interest on polymersomes because of their structural analogies with living organelles and liposomes, and their potential applications as nanosized reactors and drug delivery systems [5,6] among others [7]. Block copolymer polymersomes have revealed to be promising alternatives to phospholipid-based vesicles regarding their stealthiness [3], improved stability, and ease of functionalization. Using the versatility of polymer chemistry, various polymersomes have been designed to combine features required for drug delivery applications, such as biodegradability, ability to load hydrophilic and hydrophobic drugs, and responsiveness to biologically relevant stimuli (pH and temperature, among others) [8]. 

There are generally three main strategies to form polymersomes: To start from the bulk material in contact with water to form the final structures [9] (a “top down” approach), a “bottom up” approach starting from a completely dissolved amphiphilic block copolymer and then triggering the self-assembly process (nanoprecipitation or solvent displacement), and the formation of polymersomes during the polymerization or polymerization-induced self-assembly (PISA) process [10,11]. Among the mentioned techniques, solvent displacement is a reliable method which gathered reproducibility and control of particle size in a short time. Due to its versatility, this procedure can be applicable to a wide variety of polymers when suitable combinations of solvents are chosen [12]. For biomedical applications, the availability of stimuli-sensitive micelles or polymersomes able to control the release of drugs as a response to a slight change in pH or temperature is an appealing strategy. One class of potential candidates as both, pH- and temperature, responsive polymers, are the poly(tertiary amine-methacrylates), such as poly(*N,N*-diethylaminoethyl methacrylate) (PDEAEM) [13,14,15,16,17]. These polymers can be blockcopolymerized with a second, hydrophilic or hydrophobic polymer, to form the desired stimuli-sensitive polymer aggregates. In a previous study of our research group [17], poly(ethylene glycol)-*b*-poly(*N,N*-diethylaminoethyl methacrylate) (PEG_47_-*b*-PDEAEM_m_) copolymers were synthesized by reversible addition–fragmentation chain transfer (RAFT) polymerization. Direct dispersion in water of the diblocks resulted in polymersomes with sizes around 100 nm, depending on the lengths of the PDEAEM segment as well as the pH of the solution. These materials were used as nanoreactors for the preparation of gold nanoparticles (AuNPs) in aqueous medium. One limitation of these materials was that only spherical AuNPs could be obtained and PDEAEM contents higher than 60% required long agitation periods for aggregate preparation and generated bimodal distributions of aggregates. 

On the other hand, light-sensitive nanomaterials have recently received increasing attention for biomedical applications: photothermal therapy, photodynamic therapy and light-triggered drug delivery systems are being developed due to the high specificity and the non-invasive and low toxicity nature of the light stimulus [18,19,20,21]. Gold nanorods (AuNRs), similarly to gold nanoshells, nanostars and nanocages, show structure-dependent optical properties, with a tunable photothermal response to light [22]. In the particular case of AuNRs, two absorption bands, the transverse and longitudinal modes of the localized surface plasmon resonance (LSPR), can be observed in a typical UV/Vis absorbance spectrum; one appears at 520 nm, while the other can be tuned within the visible or near infrared (NIR) spectrum, through the aspect ratio (length divided by diameter) of the nanorod. This distinctive optical characteristic, combined with other properties such as biocompatibility and facile surface functionalization, opens up several possibilities for fascinating applications of AuNRs in the biomedical field [23,24,25,26,27]. One of the main challenges for the application of AuNRs in therapy is their proper stabilization and selective delivery to goal cells. Methods tested include their surface functionalization [28,29] and encapsulation with polymers using layer-by-layer techniques [30] and thiol groups [24]. Their encapsulation in micelles [25] and specially polymersomes [23] has been scarcely studied.

For the present study, gold nanorods with two different aspect ratios were synthesized by seed-mediated growth methods. They were encapsulated later with PEG-*b*-PDEAEM block copolymers synthesized by RAFT polymerization with macro chain transfer agents of 51 and 122 poly(ethylene glycol) repetitive units. The obtained polymersomes, containing gold nanorods and doxorubicin, were tested for their potential use as nanometric photothermal agents and drug delivery systems. The polymersomes are able to release their content as a response to changes in the pH of the environment, and also in response to temperature changes induced by the absorption of NIR laser light by the nanorods.

## 2. Materials and Methods

### 2.1. Materials

The monomer *N,N*-diethylaminoethyl methacrylate (DEAEM, Sigma-Aldrich, Toluca, México) was distilled under reduced pressure before use. Tetrachloroauric acid trihydrate (HAuCl_4_.3H_2_O), hexadecyltrimethyl-ammonium bromide (CTAB), ascorbic acid, silver nitrate (AgNO_3_), sodium borohydride (NaBH_4_), methoxy-poly(ethylene glycol) (M_n_ = 2000 g/mol and M_n_ = 5000 g/mol), *p*-dioxane (anhydrous), 4-dimethylamino pyridine (DMAP), 4,4-azobis(4-cyanovaleric acid) (ACVA), doxorubicin hydrochloride (DOX), and *N,N’*-dicyclohexylcarbodiimide (DCC) were purchased from Sigma-Aldrich, Toluca, México, and used as received. The following solvents were provided by FERMONT, Monterrey, México, and used as received: dichloromethane (DCM), tetrahydrofurane (THF), dimethyl sulfoxide (DMSO), toluene, diethylether and petroleum ether. Purification of synthetic products by column chromatography was performed using silica gel (70–230 mesh, Acros Organics, Geel, Belgium).

#### 2.1.1. Macro Chain Transfer Agent (macroCTA) Synthesis

Two different macro chain agents were synthetized using methoxy-poly(ethylene glycol) with different molecular weights (M_n_). Their M_n_ and repetitive unit number (n) were previously confirmed by ^1^H-NMR analysis, which are available in the Supplementary Materials file: M_n_ = 2276 g/mol, n = 51; M_n_ = 5400 g/mol, n = 122, (Figure S1). The synthesis of the PEG51-macroCTA was conducted as previously reported in the literature [17]. For the synthesis of the PEG_122_-macroCTA, the same synthetic protocol was adapted; details are described in the Supplementary Materials file, and their ^1^H-NMR spectra are also shown (Figure S2).

#### 2.1.2. Synthesis of Block Copolymers PEG-b-PDEAEM

The synthesis of the block copolymers was carried out as described in the literature [17]. For the present work, a series of block copolymers was prepared by using different ratios of *N,N*-(diethylamino)ethyl methacrylate (DEAEM) and the PEG_51_- or PEG_122_-macroCTAs. The synthesis of the block copolymer PEG_122_-*b*-PDEAEM_96_ is briefly described: DEAEM (1.0 g, 5.4 mmol), PEG_122_-macroCTA (0.2702 g, 0.05 mmol) and 4,4-azobis-4-cyanovaleric acid (0.0035 g, 0.0125 mmol) were dissolved in 4 mL of *p*-dioxane and mixed in a glass vial. This mixture was transferred to an ampoule. Oxygen was removed using three freeze (under Nitrogen) –thaw (under vacuum) evacuation cycles, and the ampoule was sealed with a propane torch flame under vacuum. The solution was heated to 70 °C in a mineral oil bath with magnetic stirring. At designated times (24, 36 or 48 h), the polymerization was stopped by cooling to room temperature. Then, the polymer solution was concentrated with a rotary evaporator. The mixture was dissolved with the minimum amount of DCM and precipitated with 15 mL of petroleum ether (x3). Then, the polymer was dissolved with a 2:5 DCM:ethanol solvent mixture and stirred for 30 min; later, 12 mL of cold diethyl ether was added and the sample was left undisturbed for 24 h. The white precipitate was discarded and the yellow liquid concentrated with a rotary evaporator. The final product was dried in a vacuum oven at 35 °C for 24 h. Polymerization yields were determined using the gravimetric method; in this case, 43.7% yield was obtained.

PEG_122_-*b*-PDEAEM_96_; M_n_(GPC) = 28082 g/mol, Ð = 1.22 ^1^H-NMR (400 MHz, CDCl_3_, δ, ppm): 3.99 (OCOCH_2_CH_2_N), 3.64 (CH_2_CH_2_O of the PEG chain), 2.69-2.57 (OCOCH_2_CH_2_NCH_2_), 3.38 (OCH_3_, chain end of the PEG), 1.81 (CH_2_ of the polymer backbone), 1.25 (aliphatic chain of the CTA), 1.17-0.9 (CH_3_ of the polymer backbone and NCH_2_CH_3_).

#### 2.1.3. Preparation of Nanometric Polymer Aggregates

For the preparation of the copolymer aggregates in this work, two different methodologies were used: direct dispersion in distilled water and solvent displacement or nanoprecipitation using THF/water. Direct dissolution: Consist of the solubilization of the bulk copolymer PEG_m_-*b*-PDEAEM_n_ (5 mg) in distilled water (10 mL) under magnetic stirring at 25 °C for 48 h. 

Solvent displacement or nanoprecipitation: At first, 5 mg of the copolymer was dissolved in 1mL of THF, then, 10 mL of distilled water was added dropwise to the copolymer solution under magnetic stirring. The copolymer solution was stirred in an open container for 24 h inside an extraction hood to remove the THF, allowing polymer aggregate formation.

#### 2.1.4. Gold Nanorod Synthesis

The AuNR synthesis was performed based on a previously reported methodology [24]. First, CTAB-coated gold seeds were prepared by chemical reduction of HAuCl_4_ using NaBH_4_. In a typical procedure, 0.25 mL of HAuCl_4_ solution (10 mM) was mixed with 7.5 mL of CTAB solution (100 mM) in a 20 mL vial under stirring. Then, 0.6 mL of ice-cold NaBH_4_ solution (10 mM) was injected quickly under vigorous stirring; immediately the solution turned brown-yellow, suggesting the formation of gold seeds. The gold seed dispersion was stored for 2 or 8 h before further use to allow the decomposition of excess NaBH_4_. Second, the AuNRs were prepared from gold seeds within a growth solution. Specifically, a growth solution containing 100 mL of CTAB solution (100 mM), 5 mL of HAuCl_4_ solution (10 mM), 1 mL of AgNO_3_ solution (10 mM), and 1 mL of H_2_SO_4_ (1 M) was poured into a 250-mL round-bottom flask and equilibrated at 30 °C under stirring for 30 min, and then, 0.8 mL of an ascorbic acid solution (100 mM) was injected quickly under vigorous stirring; immediately the solution became colorless. Then, 250 mL of gold seed dispersion from the first step was added under vigorous stirring for 2 min and left undisturbed for 6 h; the solution turned violet-blue. The prepared AuNRs were purified via centrifugation (12,000 rpm for 15 min) followed by dispersion in 40 mL of de-ionized water. This purification step was repeated three times. 

#### 2.1.5. Gold Nanorod Encapsulation

Two sizes of gold nanorods were coated using polymersomes: AuNR-846 and AuNR-761. For this, 2 mL of a PEG_m_-*b*-PDEAEM_n_ block copolymer solution (10 mg/mL) in DMSO was added dropwise to a AuNRs dispersion in water (10.0 mL, 0.18 mg/mL) under strong stirring. After 48 h under stirring, the dispersion was centrifuged and washed with water (14,000 rpm, 12 min) to remove excess polymer (supernatant) and then dialyzed against deionized water to remove the excess of DMSO using a 12 KDa MWCO dialysis bag from SpectraPor^®^ (four water changes every 30 min and then four changes every hour). Unloaded AuNRs precipitate inside the dialysis-bag, while encapsulated/stabilized gold nanorods were removed and quickly frozen in a acetone/dry ice bath (~−70 °C). The polymersome-coated gold nanoparticles were then freeze-dried to characterize the obtained PEG_m_-*b*-PDEAEM_n_@AuNRs by different methodologies. The AuNR loading content (LC_AuNR_) was determined by thermogravimetric analysis (TGA), subtracting the residue after measurement of the plain blockcopolymers from the residue of the AuNR loaded polymersomes. The AuNR loading efficiency (LE_AuNR_) was estimated by the mass ratio of the loaded AuNR in each polymersome to the mass of AuNR in the loading solution used. 

### 2.2. Measurements

Hydrogen nuclear magnetic resonance (^1^H-NMR) spectra were collected on a Bruker AMX-400 (Bruker Corporation, Billerica MA, USA) (400 MHz) spectrometer and are reported in ppm using tetramethylsilane (TMS) as the internal standard. The solvent used was deuterated chloroform, CDCl_3_, for all samples. 

Gel permeation chromatography (GPC) was performed on a Viscotek 305 TDA chromatograph, (Malvern, Worcestershire, United Kingdom) equipped with two T-columns in series (CLM3009: styrene divinylbenzene copolymer) and five detectors: refractive index (RI), viscosity (VISC-DP), UV-PDA and light scattering (RALS and LALS). The measurements were performed in THF at 35 °C. The conversion of the monomers to the polymer was determined by gravimetric method.

Thermogravimetric analysis (TGA) was performed on a TA-Instruments Discovery-TGA equipment (TA-Instruments, New Castle DE, USA). Measurements were performed for blockcopolymers and AuNR loaded polymer aggregates by heating under nitrogen flow from room temperature up to 600 °C at a heating rate of 10 °C/min.

Dynamic light scattering (DLS) measurements were carried out in 0.5 mg/mL block copolymer solutions at 25 °C using a Malvern Instruments Nano-ZS Nanosizer (ZEN 3690), (Malvern, Worcestershire, UK). The instrument is equipped with a helium neon laser (633 nm) with size detection between 0.6 nm and 5μm. DLS experiments were performed at the scattering angle of 90° and the distribution of sizes was calculated using Malvern Instruments dispersion technology software, based on CONTIN analysis and Stokes-Einstein equation for spheres as usual. 

UV–Vis spectra were acquired by using a UV-Vis Varian Cary 100 spectrophotometer system (Agilent Technologies, Santa Clara, CA, USA) at room temperature on AuNPs dispersions for the measurement of the localized surface plasmon resonance. In the cases where the localized surface plasmon resonance was found at higher wavelengths than 900 nm, an UV-Vis-NIR Varian Cary 5000 spectrophotometer was used for measurements in wavelengths up to 1000 nm. 

Atomic Force Microscopy (AFM) characterization of the aggregates was performed by means of a SPM 5100 atomic force microscope (Agilent Technologies, Santa Clara, CA, USA) in intermittent mode using silicon cantilevers in the 145 KHz to 160 KHz frequency range using amplitudes of 3 to 5 V. The scanner (N9520A) operation interval was 10 µm × 10 µm. Samples were prepared using concentrations of 0.05 mg/mL of the copolymers in distilled water, and a drop was placed over a mica substrate at 25 °C. Images were edited using the WSxM Develop 3.0 software from electronic nanotechnology. 

Scanning electron microscope micrographs were acquired by analytical field emission scanning electron microscope (FESEM) Jeol model JSM-7800F Prime (JEOL Ltd, Tokyo, Japan). The images were acquired in the scanning transmission electron microscopy (STEM) mode, using a bright field (BF) and a high angle annular dark field (HAADF) detector working at 25 kV. Samples were prepared by dispersing 0.5 mg of samples in 10 mL of water under stirring for 48 h. A drop of those dispersions was poured over a 300-mesh lacy carbon copper grid followed by drying in air at room temperature. Diameter and Length distribution histograms were plotted from measurements of AuNRs one by one from the FESEM images.

Transmission electron microscopy micrographs (TEM) were acquired by using a H7500 transmission electron microscope (Hitachi Co. Ltd., Tokyo, Japan) operating at an accelerating voltage of 80 kV. Samples were prepared by dispersing 0.5 mg of blockopolymers in 1 mL of water under stirring for 48 h. A drop of those dispersions was poured over a 75-mesh copper grid coated with a thin layer of carbon followed by removing excess liquid at room temperature. Afterwards, the samples were stained using a 1% uranyl acetate solution for 1 min. Diameter distribution histograms were plotted from measurements of one representative TEM image for each sample applying a Gaussian fit.

The Zeta potential (ζ) of empty, AuNR-loaded, DOX-loaded and AuNR/DOX-loaded polymersomes dispersion (0.5 mg/mL) were measured using a Malvern ZetaSizer Nano ZS instrument (Malvern, Worcestershire, UK). All the measurements were the average of three runs and performed at 25 °C and pH = 7.4 (only the empty polymersomes were measured at different pH values to estimate their isoelectric point).

#### 2.2.1. NIR Induced Heating Studies

The first light-induced heating studies of the AuNR-loaded polymersomes aqueous suspension were performed with a diode laser (808 nm, Oclaro Inc., San Jose, CA, USA) with a maximum output power of 450 mW. A 1.5-mL Eppendorf tube was filled with the PEG_m_-*b*-PDEAEM_n_@AuNRs aqueous suspension with a concentration of 0.25 mg/mL, and then fixed inside a foam fixture. The evolution of temperatures due to the laser irradiation of the aqueous suspension was monitored with a thermocouple, placed 1 cm below the liquid surface. A plain water sample was irradiated under the same conditions and was used as control. For the second irradiation study series, a Titanium-Sapphire laser oscillator (Griffin, KMLabs Inc., Boulder, CO, USA) operating at 808 nm in the continuous-wave mode (cw) was used, in this case the output laser power was varied from 100 to 200 mW. 

#### 2.2.2. Loading of Doxorubicin

Based on the methodology reported by Lecommandoux [31], for a DOX/copolymer ratio of 0.3/1 on the feed, 10 mg of block copolymers and 3 mg of DOX were dissolved on 1 mL of DMSO, then PBS at pH 7.4 was added and left under magnetic stirring for 48 h. The excess of DOX and DMSO were removed by dialysis against distilled water (500 mL, SpectraPor^®^ dialysis tubing MWCO 12–14 KDa); the external medium was renewed every 30 min for 2 h and then every hour for 4 h giving a total of eight water changes. The purified material was quickly frozen using an acetone/dry ice bath (~–70 °C) to keep the formation of ice-crystals small and then freeze-dried. The mass of DOX loaded in the polymersomes was determined by preparing a 0.5-mg/mL solution in DMSO, measuring the absorbance by UV analysis at λ_max_ = 484 nm and then quantified by using a calibration curve of DOX in DMSO (Figure S3 in Supplementary Materials file). To obtain polymersomes loaded with DOX and AuNR, the same methodology was used with the only difference that AuNR purified solution was added to the DOX/copolymer in DMSO dispersion. The loading efficiency of DOX (DLE_DOX_) and the loaded DOX content (DLC_DOX_) were calculated using the following simple equations:

DLE_DOX_ = (mass of DOX in polymersomes)/(mass of DOX in loading solution) (1)

DLC_DOX_ = (mass of DOX in polymersomes)/(mass of dry polymer) (2)

#### 2.2.3. In vitro release studies

For the controlled release studies, 1.5 mg of DOX loaded material was dispersed in 3 mL of buffer solution (pH 7.4 or 5.8) and then added to a dialysis tube (Spectra/Por^®^ MWCO: 1000 Da, diameter 10 mm, from Spectrum, Los Angeles, CA, USA). The dialysis tube was introduced into 50 mL of release medium inside an Erlenmeyer flask. The flask was poured inside a shaking bath (Shel Lab, model SWBR17, Sheldon Manufacturing, Inc., Cornelius, OR, USA) operating at 37 °C and at a shaking speed of 70 rpm. Three mL aliquots of the medium were taken out at different times and replaced by 3 mL of fresh PBS at every sampling point. The released fraction of DOX was calculated from UV measurements at λ_max_ = 484 nm and then quantified by using a calibration curve of DOX in PBS (Figure S4 in Supplementary Materials file).

For the NIR-triggered release studies, the same methodology was followed, with the exception that the dialysis tube containing the DOX-loaded PEG_m_-*b*-PDEAEM_n_@AuNRs was irradiated from the top every hour for 5 min during the first 10 h of the study.

## 3. Results and Discussion

### 3.1. Synthesis and Characterization of PEG-b-PDEAEM Block Copolymers

**Figure 1:**
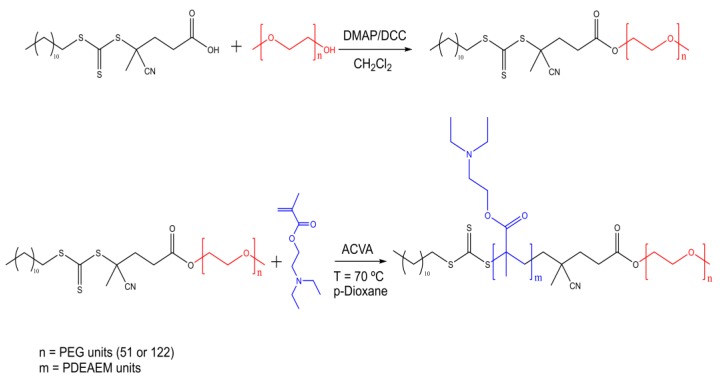
Synthetic scheme for the preparation of PEG-*b*-PDEAEM block copolymers by RAFT polymerization.

Block copolymers that contain polyethylene glycol of nominal molecular weights of 2000 and 5000 g/mol (PEG_51_ or PEG_122_) as the hydrophilic part, and PDEAEM as the pH-dependent hydrophobic part, in a proportion which allows the copolymer to be water dispersible, were prepared (Figure 1). Due to its hydrophobic characteristics, long segments of PDEAEM in the block copolymer do not allow the formation of stable aggregates in aqueous solution at neutral pH. From the literature [17], it was observed that a PDEAEM content greater than 60% for PEG_51_-*b*-PDEAEM diblocks resulted in diblocks hard to be dispersed in water and unstable aggregates that tend to precipitate. Therefore, in this investigation a series of block copolymers of different molecular weights and PDEAEM content lower than 50% were prepared by RAFT polymerization.

Table 1 shows a summary of the synthesized block copolymers. Blocks between 11,200 to 23,700 g/mol were prepared with PDEAEM contents ranging from 31 to 48%. The molecular weight for the copolymers was determined initially by GPC using a dn/dc value of 0.087 for PDEAEM in THF as reported in the literature [32]. All the prepared copolymers showed acceptable dispersity (Ð) values for a RAFT polymerization. However, in some cases, particularly for the copolymer PEG_51_-*b*-PDEAEM_47_ (P_51_D_47_), there were discrepancies between the molecular weight calculated with the RAFT equation (see Equation (3) below) and the one estimated by GPC.

This can be attributed to the fact that the dn/dc value used for the M_n_(GPC) calculation was not appropriate for the block copolymer. Equation (3) follows:

(3) $${Mn}_{\left(Calc\right)}=[(\frac{\left[M\right]}{\left[CTA\right]})x\;M_{o}x\;Conv.]+{MW}_{CTA}$$

where [*M*] is the monomer concentration, [*CTA*] is the CTA concentration, *M_o_* is the molecular weight of the monomer, Conv. is the gravimetric conversion, and *MW_CTA_* is the molecular weight of the CTA used. GPC traces for the analyzed copolymers can be observed in the Supplementary Materials file (Figure S5). 

**Figure 2:**
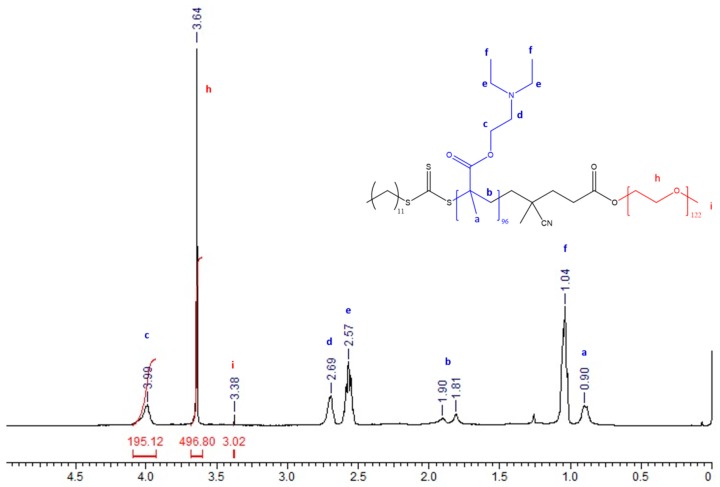
^1^H-NMR (400 Hz, CDCl_3_) spectra for the block copolymer PEG_122_-*b*-PDEAEM_96_.

The molecular weight was also obtained by ^1^H-NMR calculating the number of repetitive units of DEAEM from the integration value of the signal CH_2_–O at 4.0 ppm in relation with the signal at 3.38 ppm corresponding to the methoxy end group of the PEG-CTA (Figure S6). Results are included in Table 1 and show that in the majority of cases there is a better agreement between these values and the theoretical values as calculated using the RAFT Equation (3). The true composition of the block copolymers was also determined by ^1^H-NMR. In Figure 2, the ^1^H-NMR spectrum of the PEG_122_-b-PDEAEM_96_ copolymer is shown as an example; the following signals were observed: strong signals between 1.04 and 0.9 ppm were attributed to methyl groups of the PDEAEM segment “f” and “a”; a signal at 1.81 ppm corresponded to the methylene group in the PDEAEM backbone “b”; multiple signals in chemical shifts between 2.69 and 2.57 ppm correspond to the methylene groups next to the nitrogen atom “d” and “e”; a singlet at 3.38 ppm corresponds to the methoxy terminal group of the PEG “i”; a signal at 3.64 ppm corresponds to the methylene groups in the PEG repetitive units “h”; and a signal at 4.0 ppm was attributed to the methylene group next to the oxygen atom in PDEAEM “c”. A signal that can be attributed to the CTA can be observed at 1.25 ppm corresponding to the aliphatic chain (ten CH_2_ units). For this particular block copolymer, the true composition is 44% DEAEM and 56% EG units as calculated using the integration of the methylene groups in the PEG repetitive units at 3.64 ppm in relation to the integration of the signal at 4.0 ppm corresponding to the DEAEM methylene group next to oxygen. The NMR spectra for the other block copolymers can be found in the Supplementary material file (Figure S6).

### 3.2. Self-assembly Studies of the PEG-b-PDEAEM Copolymers

**Figure 3:**
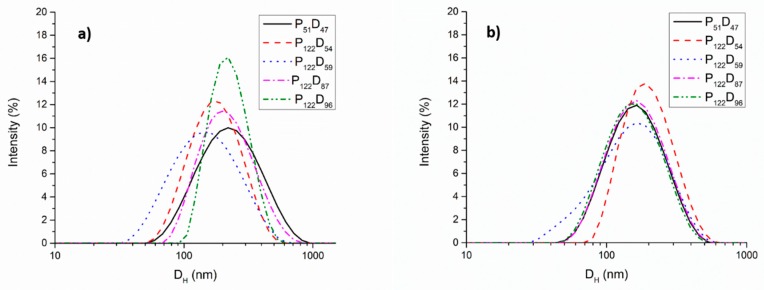
DLS distribution of sizes for the polymer aggregates obtained by: (**a**) direct dispersion in water, and (**b**) solvent exchange or nanoprecipitation.

Dynamic light scattering measurements were performed on the polymer aggregates obtained from the synthetized copolymers in water using two different preparation techniques. For the copolymer aggregates prepared by direct dispersion of the block copolymers in distilled water, all the solutions were mixed under magnetic stirring for 48 h before the analysis. All the copolymers were dispersible at concentrations of 0.5 mg/mL or lower and the pH of the solutions prior to the DLS analysis was between 7.4 and 7.7 depending on the PDEAEM content of the copolymer. Considering that the pK_a_ of the PDEAEM is 7.4 [33], at these pH values, the amine groups in the PDEAEM segments of the copolymer are only partially protonated, rendering the PDEAEM segment hydrophobic behavior, and allowing the possible formation of aggregates stabilized by the hydrophilic PEG segments. As observed in Figure 3a, all the copolymers exhibited monomodal distributions of sizes with hydrodynamic diameters of around 200 nm and polydispersities of around 0.3. In the case of aggregates obtained by solvent exchange, lower sizes and narrower distributions of sizes were observed. This may result from the fact that the amphiphilic copolymers are first homogeneously dissolved in THF and subsequently mixed with water, allowing the formation of a more stable and compact membrane for the newly formed polymeric vesicles (polymersomes) as compared to the ones obtained by direct dispersion in water of the strong hydrophobic block copolymers. The size distributions for the copolymer aggregates obtained by solvent exchange are shown in Figure 3b.

In Table 2, a summary of the D_H_ values for the polymer aggregates obtained by direct dispersion and solvent exchange, and the corresponding diameters (D) observed by AFM, are included. The extended chain lengths for the PEG-*b*-PDEAEM block copolymers were calculated for comparison purposes. For this, the number of units in the block copolymer were taken and a carbon-carbon extension in SP_3_ hybridization state for the extended PDEAEM segment (end to end distance <h^2^>^0.5^) and the Flory approximation for the random coiled PDEAEM in tetha solvent, were considered as described previously [17]. For the PEG-block, using the reported value <h^2^>^0.5^ = 3.21 ± 0.5 nm for a polyethylene oxide polymer of 36 units [34], a simple scaling to a PEG of 51 and 122 units results in <h^2^>^0.5^ values of 4.55 nm and 10.88 nm respectively. The end-to-end distance of the block copolymers studied range from 7.63 nm to 17.5 nm (coiled PDEAEM) to 16.14 nm to 35.26 nm (fully extended PDEAEM). Comparing these values with the D_H_ observed by DLS, regardless of the preparation technique used for the copolymer aggregates, a double layer aggregate such as a polymersome morphology is suspected, since a simple spherical micelle would have a diameter of 15.3 to 32.3 nm for the PEG_51_ copolymer and of 29.3 to 70.5 nm for the PEG_122_ copolymers (two times the end-to-end distance of the polymer chains).

**Figure 4:**
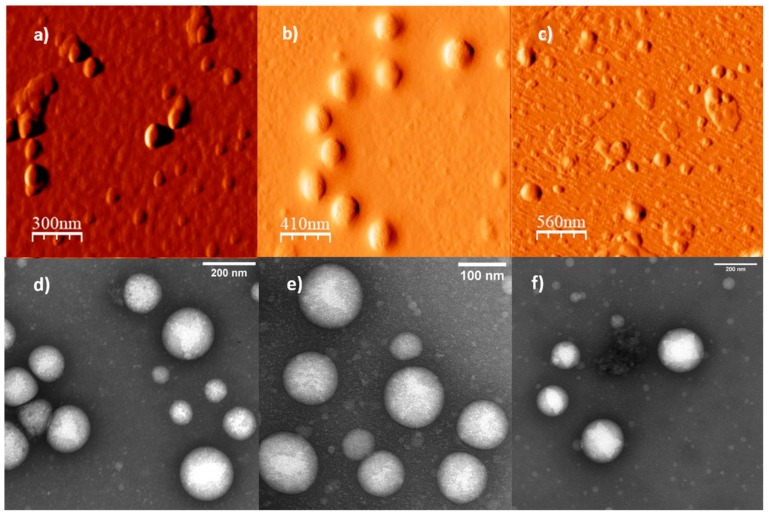
AFM and TEM micrographs for some aggregates obtained by solvent exchange for the copolymers: (**a**) AFM image of P_51_D_47_, (**b**) AFM images of P_122_D_54_, (**c**) AFM images of P_122_D_96_, (**d**) TEM image of P_51_D_47_, (**e**) TEM image of P_122_D_54_, and (**f**) TEM image of P_122_D_96_. AFM Images obtained on mica substrate by tapping mode and TEM images with uranyl acetate stained samples on copper grids.

To confirm the morphology of the suspected polymersomes, AFM and TEM analysis were performed on the polymer aggregates obtained by the solvent exchange methodology. The obtained micrographs are presented in Figure 4. All the obtained aggregates possess semispherical shapes with measured diameters comparable to that obtained by DLS, observing a greater standard deviation for the P_122_D_96_ aggregates. TEM micrographs show a clearer contrast in the middle of the aggregates with a distinctly darker contrast in the periphery. This is a result of the preferential staining of the DEAEM units in the aggregates which are concentrated in the periphery of the aggregates, supporting the double layer formation with a water core (vesicle type polymersomes).

### 3.3. pH and Temperature Sensitive Behavior of the PEG-b-PDEAEM Aggregates

**Figure 5:**
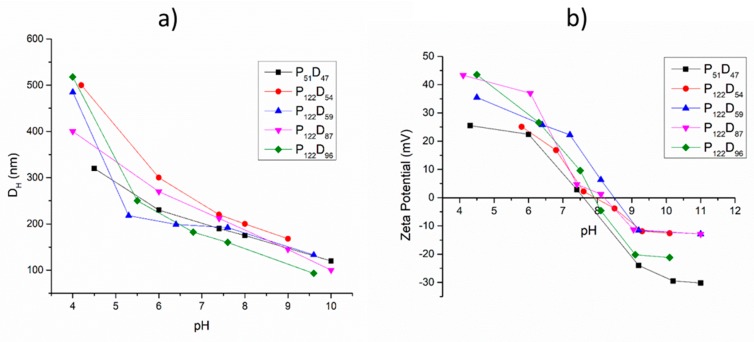
pH-sensitive behavior for aggregates prepared from block copolymers: (**a**) Hydrodynamic diameters obtained by DLS (0.5 mg/mL dispersion) and (**b**) Zeta potential measurements on polymer aggregates.

To study the pH-sensitive behavior of PEG-*b*-PDEAEM, 0.5 mg/mL dispersions of polymersomes in distilled water were used. The pH of the solution was adjusted by adding HCl or NaOH solutions. Figure 5a shows the pH-sensitive behavior of the polymersomes generated from all the prepared copolymers. An increase of the polymersome diameter at pH values lower than 7 can be observed due to the electrostatic repulsion between the protonated amine groups on the PDEAEM segments. Increases in the D_H_ values from 144 to 185% are observed at a pH value of 4 (in the case of the copolymers P_122_D_96_, P_122_D_59_ and P_122_D_54_), indicating the destruction of the original polymersome and the formation of larger aggregates (Figure S8 in the Supplementary material file). In the biologically significant pH interval from 5.5 to 8, all the polymersomes are stable, which makes them great candidates for drug delivery applications. The pH-sensitive behavior of the polymersomes was also reflected in the zeta potential analysis (Figure 5b), observing an increase of the total positive charge as the pH value decreased and a decrease in the zeta potential value when the tertiary amine groups of the PDEAM segments were deprotonated at higher pH values [33].

At the physiological pH value of 7.4, the polymersomes presented positive zeta potential values of +0.5 to +25 mV depending on the PDEAEM content. These value results are appropriate for a possible cell internalization of the aggregates.

**Figure 6:**
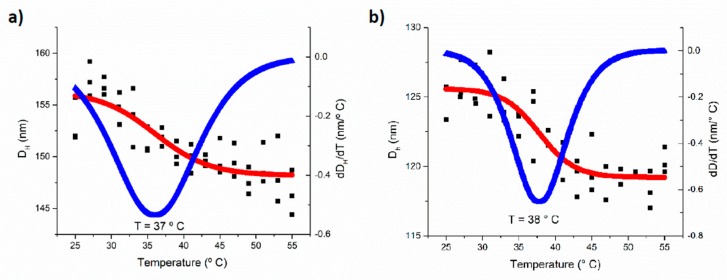
Temperature sensitivity of polymer aggregates at a pH 7.4: (**a**) aggregates formed by P_122_D_54_ and (**b**) aggregates formed by P_122_D_87_.

The temperature sensitivity of the obtained polymersomes was confirmed by DLS measurements, one of several methods used to determine the LCST [35]. Aqueous dispersions of polymersomes (0.5 mg/mL, pH adjusted at 7.4) were used; these dispersions were studied using a heating ramp of 2 °C/5 min, from 25 to 55 °C. As seen in Figure 6, a slight contraction (5% in the D_H_) of the polymersomes due to the temperature increase was observed. Taking the numerical derivate of the data (blue curve), a phase transition temperature of 37–38 °C was calculated. The same behavior was observed for all the copolymers. Since it is known that the temperature-sensitive behavior depends on the hydrophobic–hydrophilic balance of the copolymers [36], it is expected that the pH of the aqueous environment will strongly affect this behavior for the title copolymers. In fact, it has been reported that for PDEAEM nanogels, the LCST is shifted from 38 °C to 65 °C when the pH of the aqueous environment changes from pH 7.3 to pH 6.7, and at pH values lower than 6.5 there is no LCST [37]. The PEG-*b*-PDEAEM block copolymers employed in this study did not show a transition temperature in the range between 25 and 55 °C when studied at a pH of 5.8—a pH where the ionization of the DEAEM units is strong.

### 3.4. Gold Nanorod Synthesis

Gold nanorods were synthetized by a seed-mediated growth method. Varying the age of the gold seeds, two different types of gold nanorods with different aspect ratios where synthetized. 

**Figure 7:**
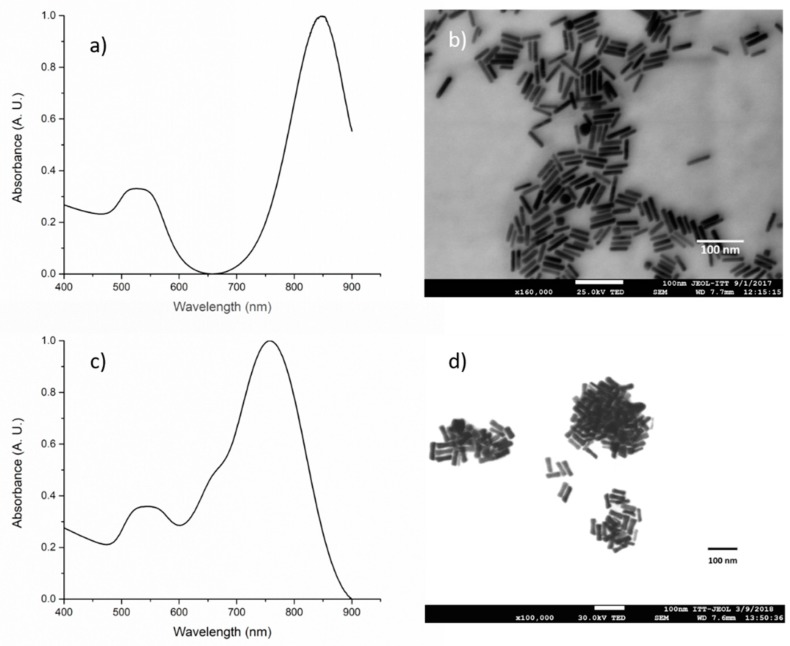
Gold nanorod’s characterization: (**a**) UV-Visible spectrum, (**b**) FE-SEM micrograph for the gold nanorods synthetized from 2-h-aged gold seeds, (**c**) UV-Visible spectrum, and (**d**) FE-SEM micrograph for the gold nanorods synthesized from 8-h-aged gold seeds.

Seeds aged for 2 h lead to gold nanorods with a smaller width and, as a consequence, a higher aspect ratio than the ones obtained with the 8-h-aged seeds. For the gold nanorods prepared using 2-h-aged seeds (AuNR-846), the measured absorbance spectrum, right after the synthesis, is shown in Figure 7a. The spectrum reveals a longitudinal Surface Plasmon Resonance (LSPR) band at 846 nm. By FE-SEM microscopy observation, an average size of 7.33 ± 1.16 nm in width and 42.12 ± 6.6 nm in length was estimated, which results in an aspect ratio of about 5.7. In the case of the gold nanorods synthetized using 8-h-aged seeds (AuNR-761) a LSPR band at 761 nm is observed (Figure 7c). By FE-SEM microscopy observation, an average diameter of 12.13 ± 2.3 nm in width and 49.38 ± 6 nm in length was estimated; therefore, the aspect ratio resulted to be 4.07. These results coincide with the reports from C.J. Murphy et al. [38] which demonstrated that a higher aspect ratio of AuNRs results in a shift of SPR to higher wavelengths.

### 3.5. Gold Nanorod’s Encapsulation

**Figure 8:**
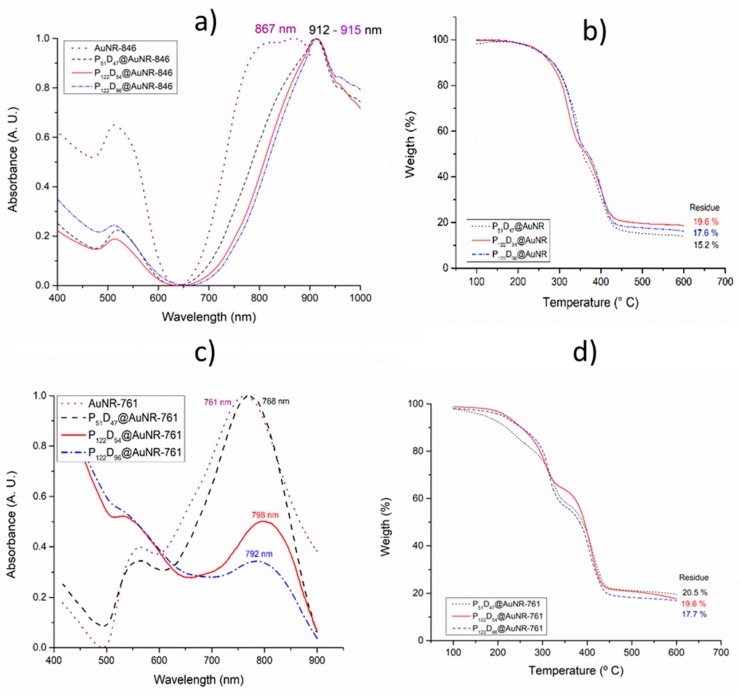
Gold nanorod filled polymersome’s characterization: (**a**) UV-Visible spectrum, and (**b**) TGA thermograms for the gold nanorods synthetized from 2 h aged gold seeds; (**c**) UV-Visible spectrum, and (**d**) TGA-thermograms for the gold nanorods synthesized from 8 h aged gold seeds.

By modifying the methodology reported by Zhong et al. [25], the synthetized gold nanorods were encapsulated on selected block copolymers, observing in all cases a displacement of the longitudinal LSPR band to higher wavelengths (Figure 8). In the case of gold nanorods with a LSPR longitudinal band of 846 nm, right after the purification it was observed that the LSPR band was displaced to 867 nm (AuNR-846). 

**Figure 9:**
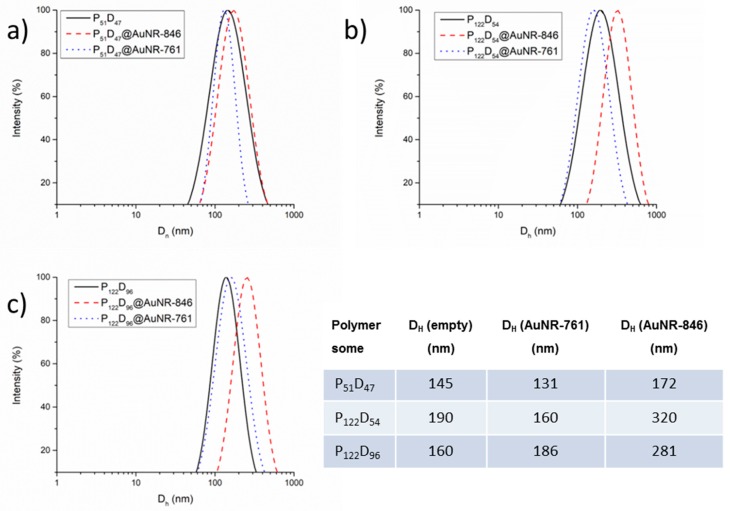
Comparison between the D_H_ of empty polymersomes and AuNR-846/AuNR-761 loaded polymersomes in PBS at pH 7.4: (**a**) from P_51_D_47_, (**b**) P_122_D_54_, (**c**) and P_122_D_96_.

This is probably due to agglomeration between the gold nanoparticles after the CTAB removal and a possible increase of the local refractive index. For the encapsulated nanorods, the LSPR longitudinal bands were further displaced to values around 912 and 915 nm (Figure 8a). By TGA, the block copolymers show two decomposition steps, the first one between 200 and 350 °C and the second one between 350 and 450 °C (thermograms of the block copolymers without AuNRs in Figure S9, in the Supplementary material file). After heating to 600 °C, the obtained residues for the copolymers P_51_D_47_, P_122_D_54_ and P_122_D_96_ are between 4.3 and 5.5%. By the encapsulation of AuNR-761, also a slight displacement in the LSPR longitudinal band of the gold nanorods was observed as a result of the AuNR agglomeration after the CTAB removal. For the encapsulated nanorods, the LSPR longitudinal band was displaced only minimally to a maximum of 798 nm (Figure 9c). Unlike the high wavelength values obtained for the LSPR of the copolymer encapsulated AuNR-846 (~910 nm), the resonances centered around 768–798 nm are more suitable to obtain a good photothermal efficiency by irradiating with a wavelength of 808 nm. The encapsulated AuNR content in the polymersomes was determined by TGA analysis, subtracting the residue from the block copolymers to those of the PEG_m_-*b*-PDEAEM_n_@AuNR materials. In the case of AuNR-761, the content in the polymersomes range from ~12 to ~16% by weight (Figure 8d) while the loading efficiency of AuNR ranges from 43 to 45% (data in Table 3). 

The encapsulation of AuNRs in the polymersomes has a side effect—a change in the overall diameter of the polymersomes. Figure 9 shows the distribution of sizes by DLS for the empty polymersomes as compared to the AuNR-761 and AuNR-846 filled polymersomes. For the block copolymer containing a short PEG segment (P_51_), the size changes are small (~19%); however, for the block copolymers with a larger PEG segment (P_122_), the size increase goes up to ~75%, suggesting that the introduction of AuNRs alters the aggregation equilibrium of block copolymers resulting in larger polymersome formation in this case. 

Interestingly, this effect is larger for the AuNR-845 loaded polymersomes, probably because these AuNRs are also larger in size making it more difficult to be encapsulated inside the polymersomes.

### 3.6. Photothermal Heating under NIR laser Irradiation

By irradiating the AuNRs with NIR laser light (wavelength of 808 nm), a heating of the surroundings of the AuNRs is expected to appear. Depending on the amount of generated heat by the irradiated AuNRs, the temperature reached could lead to one of the following results: at temperatures higher than 43 °C, protein denaturalization by hyperthermia may occur [39,40]; while a smaller temperature increment could lead to a thermosensitive response of the PDEAEM blocks of the PEG-*b*-PDEAEM polymersomes, resulting in squeezing out the encapsulated water from the polymersome; this effect could be used to enhance drug delivery [41]. An interesting aspect of combining a nanocarrier AuNRs with a chemotherapeutic agent, like doxorubicin (DOX), is an expected synergic therapeutic effect: The hyperthermia generated by the NIR-Irradiation can sensitize the cancer cells, promoting the drug delivery directly into the tumors, increasing the therapeutic effects of the separate photothermal and chemotherapy [42,43].

**Figure 10:**
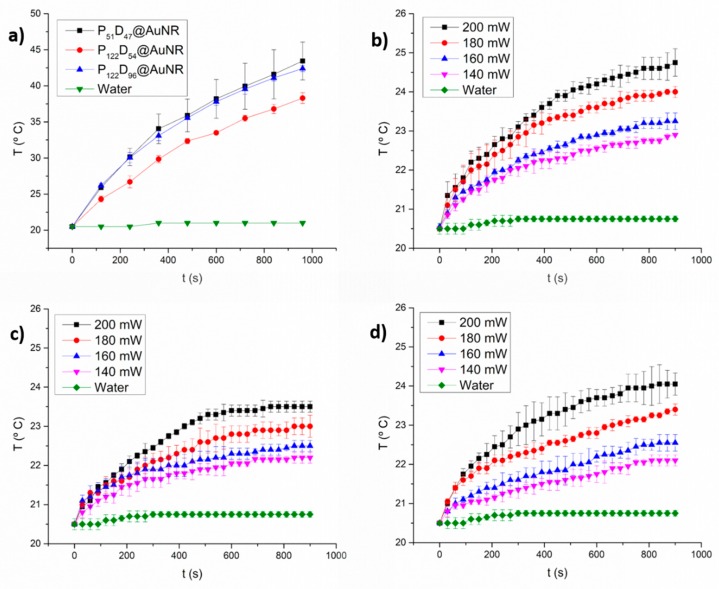
Time-dependent temperature increase due to NIR laser irradiation (808 nm) of PEG_n_-*b*-PDEAEM_m_@AuNR dispersions in distilled water (0.5 m/mL): (**a**) at 450 mW, (**b**) PEG_51_-*b*-PDEAEM_47_@AuNR varying the laser output power, (**c**) PEG_122_-*b*-PDEAEM_54_@AuNR varying the laser output power and (**d**) PEG_122_-*b*-PDEAEM_96_@AuNR varying the laser output power.

As seen in Figure 10, with a laser output power of 450 mW, water did not show an increment in temperature during the 15 min of irradiation, because the water molecules do not absorb light at 808 nm. However, irradiation of the polymersome-encapsulated AuNRs, increments the temperature of the medium between 17 and 24 °C above room temperature (Figure 10a). Differences in AuNRs concentrations are expected to be rather small among samples, as estimated from the thermogravimetric analysis data (Figure 8d). 

At lower laser output powers, temperature increments between 5 °C (200 mW) and 1.5 °C (140 mW) are reached after 15 min of irradiation (Figure 10b–d). Although the measurements represent only macroscopic spatial averages and are not necessarily representative of the local temperature at the micro scale, it is possible that the temperature increments with laser output powers lower than 200 mW may be too small to obtain a significant response for NIR-triggered drug release experiments.

### 3.7. Load and Controlled Release of DOX from PEG-b-PDEAEM Polymersomes

#### 3.7.1. DOX Loading

The DOX-loaded PEG-*b*-PDEAEM polymersomes (with and without AuNR) were obtained by nanoprecipitation or solvent exchange, an effective bottom up strategy which allows the encapsulation of both hydrophobic and hydrophilic drugs [44]. To a DMSO solution of DOX and the PEG-*b*-PDEAEM block copolymers, PBS with a pH value of 7.4 was added to allow the formation of the drug-loaded polymersomes. Given the pH-sensitive properties of the empty PEG-*b*-PDEAEM polymersomes, and knowing from the literature that DOX possess a pK_a_ value of 8.4 for the daunosamine-NH_3_ group [45], it is expected that at pH 7.4, the amine moieties present in both components are only slightly protonated, allowing the encapsulation of DOX in the polymersome. As seen in Table 3, DOX-loading efficiencies between 47 and 52% and DOX loading contents between 10.8 and 12% for the polymersomes without AuNR were obtained; these parameters are better (almost two times higher) than those reported for similar polymersomes in the literature [46]. These percentages decrease slightly when AuNR are loaded in the polymersomes. 

Drug release experiments were conducted at two different pH values (for the DOX and the AuNR-DOX containing polymersomes). Additional irradiation of the AuNR containing polymersomes was performed every hour for 5 min with a wavelength of 808 nm. Free DOX, dissolved in PBS at pH 7.4 and treated under the same conditions as the test materials, was used as a control for each experiment. 

#### 3.7.2. pH Triggered DOX Release

**Figure 11:**
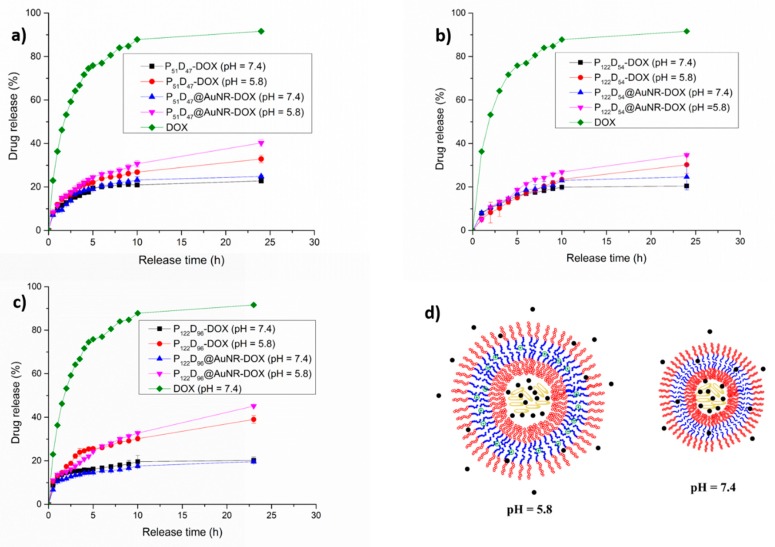
In vitro release profiles for DOX and AuNR-DOX loaded polymersomes at different pH values: (**a**) P_51_D_47_ polymersomes, (**b**) P_122_D_54_ polymersomes, (**c**) P_122_D_96_ polymersomes and (**d**) schematic representation of polymersomes at pH values of 5.8 and 7.4.

A pH-controlled DOX release has been a goal in different investigations using nanocomposites over the last years [43,46,47,48,49]; since it is known that the tumor pH and endosomal pH is slightly acidic, the pH change can be used as an on-demand drug release trigger. The studies of the pH sensitivity of the obtained polymersomes showed that at pH values lower than 7, an increment in the D_H_ occurs due the electrostatic repulsion between the protonated amine groups on the PDEAEM segments. This should not change much if the AuNRs are encapsulated (see Figure S10 in the Supplementary Material File). For the P_51_D_47_ polymersomes (Figure 11a), during the first 6 h, 20% of the loaded DOX is released at pH 7.4. From this time on, there is only a slight increase in the release rate (22%) up to 24 h; however, when AuNRs are included, there is a small increase in the rate of release already after 10 h. At pH 5.8, the effect of the pH-sensitive behavior of the polymersomes on the rate of release is observed already after 2 h, reaching a 33% release rate in 24 h. Another interesting observation is that, although the polymersomes that do not contain AuNR accept a higher load of DOX, the polymersomes with AuNR at pH 5.8 show a higher DOX release rate, reaching 40.3% at 24 h. For the P_122_D_54_ polymersomes (Figure 11b), a similar behavior is observed; however, for the P_122_D_96_ polymersomes (Figure 11c), the pH-sensitive behavior is more evident, while at a pH of 7.4, only 20% of the loaded DOX is released; at a pH of 5.8, 39% of the loaded DOX is released when no AuNR are incorporated. In the case in which AuNR are also loaded, the released DOX at 24 h reached a value of 45%. Since at a pH value of 5.8 a plateau was not reached during the first 24 h of the release experiment, the experiment was continued for 3 more days taking a sample every 24 h. The released DOX reached 49.6% in 48 h, 52.2% in 72 h and 54.4% in 96 h. The effect of the AuNR accelerating the release rate of DOX in both types of polymersomes may result from steric destabilization of the polymersome double layer given the large size and elongated form of the AuNRs. In Figure 11c, a schematic representation is shown of an expanded polymersome as a result of the electrostatic repulsion between the protonated amine groups on the PDEAEM blocks, creating channels for the release of the encapsulated DOX in the media, channels that are not present at pH = 7.4 where the protonation of PDEAEM is only minimal.

#### 3.7.3. NIR-irradiation Triggered DOX Release

**Figure 12:**
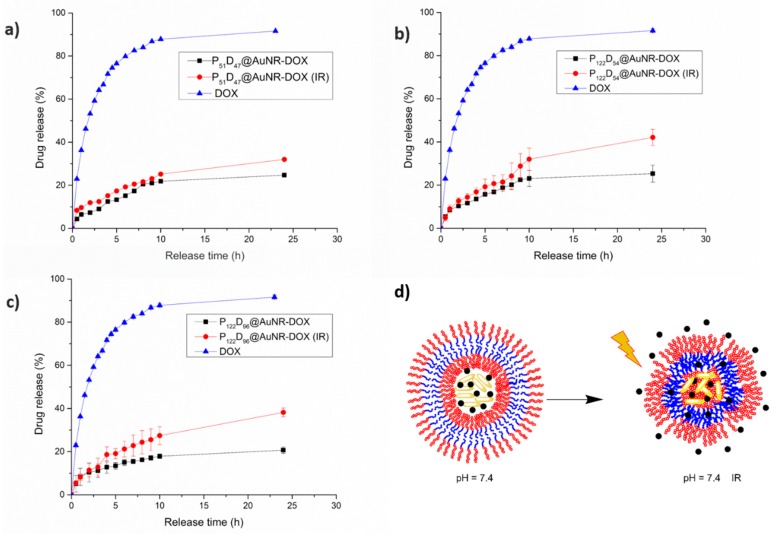
In vitro release profiles for DOX and AuNR-DOX-loaded polymersomes at pH 7.4. Effect of NIR irradiation: (**a**) P_51_D_47_ polymersomes, (**b**) P_122_D_54_ polymersomes, and (**c**) P_122_D_96_ polymersomes. (**d**) Model of polymersomes behavior.

From the NIR irradiation temperature increment studies, it was confirmed that the temperature reached after 5 min of irradiation with a laser output power of 450 mW, is high enough to allow for a thermosensitive response of the PEG-*b*-PDEAEM blocks in the polymersomes. The temperature-driven contraction of the polymersome may result in squeezing out the encapsulated DOX, nevertheless it is important to note that the change on the D_H_ of the polymersome due to a temperature change is lower than the observed pH-change. As seen in Figure 12a, for the P_51_D_47_ polymersomes there is a slight increment on the released DOX at pH 7.4 due the NIR-irradiation during the first 10 h of the experiment. After 24 h of the experiment, the difference between the irradiated and the non-irradiated samples is about 10%, reaching a DOX release of 32%, which is comparable with the DOX release at a pH value of 5.8 without NIR irradiation (Figure 11a). For the P_122_D_54_ polymersomes (Figure 12b), during the first 8 h of the drug release experiment, the difference between the NIR-irradiated and non-irradiated polymersomes is very small. At the 9th hour, the release is boosted from 24 to 29%, reaching a 42% DOX release at 24 h, as contrasted with the 25% reached by the non-irradiated system. In the case of the P_122_D_96_ polymersomes (Figure 12c), an important difference between the release profiles of the NIR-irradiated and non-irradiated systems is observed. For the irradiated system, the DOX release is boosted from 13 to 19% at the 4th hour, while for the non-irradiated system, the DOX release only increases from 11 to 13%. During the first 10 h of the experiment, the DOX release for the non-irradiated system increases pretty slowly, reaching an 18% DOX release; while for the irradiated system, at the same time a 27% of DOX release is reached. Unlike the P_51_D_47_ polymersome, for the P_122_D_96_ polymersome, there is an important difference between the amounts of released DOX due the NIR-irradiation at 24 h, being 38 and 45% respectively. In Figure 12d, a schematic representation is shown of the non-irradiated polymersome and the collapsed (irradiated) polymersome as a result of a thermosensitive response due to the temperature increment of the media produced by the NIR irradiated AuNRs, releasing more DOX.

#### 3.7.4. Combined pH and NIR-irradiation Triggered Release

**Figure 13:**
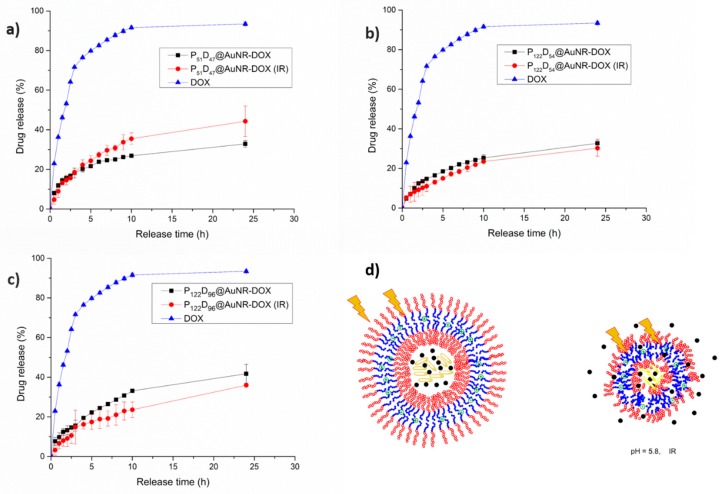
In vitro release profiles for DOX and AuNR-DOX-loaded polymersomes. Effect of NIR irradiation at a pH value of 5.8: (**a**) P_51_D_47_ polymersomes, (**b**) P_122_D_54_ polymersomes, (**c**) P_122_D_96_ polymersomes. (**d**) Model of polymersomes behavior.

While the increment in the released fraction of DOX by effect of the change to a lower pH value in the medium is due to the increment in size and permeability of the polymersome, the observed increment of released DOX due to the NIR irradiation of AuNR-containing polymersomes is a result of a contraction because the phase transition temperature of the blockcopolymers is surpassed. So what would be the effect on DOX release of a combination of pH-change with NIR-irradiation? In Figure 13, it is observed that the combination of both effects has a different response for each tested polymersome. In the case of the P_51_D_47_ polymersomes (smaller, with a balance of hydrophilic PEG and pH-ionizable PDEAEM units), the NIR-irradiated system exhibits a higher rate of release from the 5th hour; this could be attributed to the fact that the protonation of the amine groups at the pH = 5.8 plus the NIR-irradiation could lead to destabilization of the poymersome, allowing a higher DOX release. However, for the P_122_D_54_ and P_122_D_96_ polymersomes (larger, with higher hydrophilic PEG content than pH-ionizable PDEAEM units), the amount of released DOX is equal or even lower for the irradiated systems as compared to the non-irradiated ones. These observations could be attributed to the following. First, the partially protonated polymersomes have a larger PEG segment and are more stable at pH 5.8. On the other hand, at pH values lower than 7.4, the transition temperature of PDEAEM polymers tends to increase to higher values [34]. This means that the temperature-driven contraction of the polymersomes cannot be reached with the irradiation levels employed.

## 4. Conclusions

Five PEG-*b*-PDEAEM block copolymers were prepared using PEG-macro chain transfer agents containing 51 and 122 units of PEG, changing the PDEAEM block size by RAFT polymerization. The PDEAEM content in the block copolymers was changed from 31% to 44% for the copolymers containing the PEG_122_-macroCTA and was 48% for the PEG_51_ copolymer; this resulted in different sizes (D_H_ from 100 to 250 nm) for the obtained aggregates. The polymer aggregates were prepared by two different techniques, observing that the ones obtained by solvent exchange had lower diameters and narrower distributions of sizes. By AFM, the spherical morphology of the aggregates was confirmed and calculations suggested that polymersomes were obtained. These polymer aggregates were used to encapsulate gold nanorods of different sizes, observing that by the polymer coating the longitudinal surface plasmon resonance band was shifted to higher wavelengths (from 30 to 50 nm approximately). From the prepared polymer@AuNRs, the best candidates for photothermal applications were the PEG_m_-*b*-PDEAEM_n_@AuNR-761 systems, because their LSPR band was close to 800 nm and they can be efficiently excited by a NIR laser. 

The obtained PEG_m_-*b*-PDEAEM_n_@AuNR-761 from three different block copolymers (P_51_D_47_, P_122_D_54_ and P_122_D_96_) contained 12 to 15 wt% of AuNRs and was further successfully loaded with 10–12 wt% of the anti-cancer drug doxorubicin to obtain nanometric drug delivery systems with triggered drug release under pH changes and NIR laser activation. The mechanisms of the enhanced release due to the tested stimuli are completely different: By decreasing the pH of the media, the polymersome suffers an increment on the D_H_, increasing its membrane permeability, while by NIR-irradiation the heat generated by the encapsulated AuNRs leads to a D_H_ decrease on the polymersomes, squeezing out the trapped DOX. By combining both stimuli, for the P_51_D_47_ polymersome, an increase on the released DOX fraction is observed, which is attributed to the fact that by having a smaller PEG segment, the polymersome double layer is not very stable against possible expansion/contraction events, allowing a faster release of the entrapped DOX. In the case of P_122_D_54_ and P_122_D_96_ polymersomes, with a longer PEG segment and lower PDEAEM contents, the overall stability of the polymersomes is higher, even at a high degree of protonation of PDEAEM units (at pH = 5.8). These larger, more stable polymersomes are good candidates for pH-triggered drug delivery or NIR-irradiation triggered drug delivery at pH 7.4; however, a synergy by combining both effects was not observed. In future studies, the developed PEG_m_-*b*-PDEAEM_n_@AuNR-761 nanomaterials will be characterized more thoroughly, and their efficacy for the treatment of some cancer cell lines will be tested.

## Figures and Tables

**Table 1:** RAFT polymerization of DEAEM in p-dioxane at 70 °C for 48 h using PEG_51_- and PEG_122_-macroCTAs as RAFT agents.

Sample ^a^	M:macroCTA:I	Yield ^b^ (%)	M_n (CALC)_^c^ (g mol^−1^)	M_n (GPC)_^d^ (g mol^−1^)	Ð ^d^	M_n (NMR)_^e^ (g mol^−1^)	PEG:PDEAEM ^e^ (Molar ratio)
P_51_D_47_	432:8:1	51.7	12408	21020	1.11	11283	52: 48
P_122_D_54_	432:6:1 ^f^	38.9	19038	19619	1.35	15807	69: 31
P_122_D_59_	432:4:1 ^f^	39.1	25708	24431	1.14	16734	67: 33
P_122_D_87_	432:6:1	40.0	19038	23187	1.03	22047	58: 42
P_122_D_96_	432:4:1	43.7	25708	28082	1.22	23715	56: 44

^a^ The subscript numbers represents the number of repeating units of each PEG (P) and PDEAEM (D) block estimated by using ^1^H-NMR (400 MHz); ^b^ Determined by gravimetry; ^c^ Molecular weight calculated from the RAFT polymerization Equation (3) assuming a 100% conversion; ^d^ Molecular weight and dispersity (Ð = M_w_/M_n_) values determined by GPC using dn/dc = 0.087 mL/g [33]; ^e^ Determined by 1H NMR (400 MHz); ^f^ Polymerized for 36 h.

**Table 2:** Dynamic light scattering and microscopy analysis for polymer aggregates (sizes in nm).

Sample ^a^	PEG:PDEAEM ^a^	D_H_ (direct dispersion) ^b^	D_H_ (solvent exchange) ^b^	D (AFM)	D ^c^ (TEM)	n_PDEAEM_^a^	L (nm) ^d^
P_51_D_47_	52:48	208	100	109.3 ± 13.8	119.5 ± 7.4	46	7.63–16.14
P_122_D_54_	69:31	197	150	206.7 ± 26.5	105 ± 3.7	54	14.65–24.59
P_122_D_59_	67:33	140	180	163.3 ± 42.7	-	59	14.95–25.87
P_122_D_87_	58:42	217	120	122.0 ± 18.6	-	87	16.88–32.98
P_122_D_96_	56:44	238	115	173.4 ± 44.0	137.6 ± 9.6	96	17.50–35.26

^a^ Calculated by ^1^H-NMR analysis (400 MHz) from the integration value of the signal CH_2_-O at 4.0 ppm (DEAEM) in relation with the signal at 3.65 ppm corresponding to the known number of methylenes (CH_2_CH_2_-O) of the repeating unit in the PEG-CTA (see Figure S1 in Supplementary Material file). ^b^ Obtained by dynamic light scattering. ^c^ Statistics of sizes in Figure S7 in Supplementary material file. ^d^ Calculated by using the equations: <h^2^>^0.5^ = n_PDEAEM_*(0.254) and D_h_ = 2*(0.665/√6)*<h^2^>^0.5^ for the PDEAEM segment extension, 4.46 nm for the PEG_51_, and 10.88 nm for the PEG_122_.

**Table 3:** DOX loading efficiency (DLE_DOX_) and DOX loading content (DLC_DOX_) and equivalent values for AuNR loadings calculated for the obtained polymersomes.

Polymersome	DLE_DOX_ (%)	LE_AuNR_^a^ (%)	DLC_DOX_ (%)	LC_AuNR_^b^
P_51_D_47_	52.0	-	12.0	-
P_51_D_47_@AuNR-761	47.4	44.7	10.9	16.1
P_122_D_54_	46.7	-	10.8	-
P_122_D_54_@AuNR-761	44.3	42.5	10.2	15.3
P_122_D_96_	48.3	-	11.1	-
P_122_D_96_@AuNR-761	44.7	33.8	10.3	12.2

^a^ Loading efficiency of AuNR (LE_AuNR_) as determined by Equation (1) but using data from AuNR. ^b^ Loading content of AuNR (LC_AuNR_) as determined by TGA analysis, subtracting the residue from the block copolymers to those of the PEG_m_-*b*-PDEAEM_n_@AuNR materials.

## Supplementary Materials

The following are available online at https://www.mdpi.com/2073-4360/11/6/939/s1, Figure S1: ^1^H-NMR spectra for Sigma Aldrich^®^ methoxy-Poly(ethylene glycol) of (a) 2000 Da and (b) 5000 Da, Text describing of the Synthesis of the PEG_122_-macroCTA in detail, Figure S2: ^1^H-NMR (400 MHz, CDCl_3_) spectra for (a) CTA: 4-cyano-4 (dodecylsulfanylthiocarbonyl) sulfanyl pentanoic acid, (b) PEG_51_-macroCTA, and (c) PEG_122_-macroCTA, Figure S3: (a) UV-Vis spectra of DOX in DMSO at different concentrations (0–100 μg/mL) and (b) Calibration curve of DOX in DMSO at 484 nm, Figure S4: (a) UV-Vis spectra of DOX in PBS at different concentrations (0-30 μg/mL) and (b) Calibration curve of DOX in DMSO at 484 nm, Figure S5: GPC traces (RALS-detector) of the copolymers P_51_D_47_ (PEG_51_-*b*-PDEAEM_47_), P_122_D_54_ (PEG_122_-*b*-PDEAEM_54_), P_122_D_59_ (PEG_122_-*b*-PDEAEM_59_), P_122_D_87_ (PEG_122_-*b*-PDEAEM_87_) and P_122_D_96_ (PEG_122_-*b*-PDEAEM_96_), Figure S6: ^1^H-NMR spectra (400 MHz, CDCl_3_) of synthetized block copolymers: (a) P_51_D47, (b) P_122_D_54_, (c) P_122_D_59_, (d) P_122_D_87_, Figure S7: Statistics of TEM measurements of polymer aggregates: (a) P_51_D47, (b) P_122_D_54_, (c) P_122_D_96_, Figure S8: DLS distribution of sizes for the P_122_D_96_ polymer aggregates obtained by nanoprecipitation in water at different pH values, Figure S9: TGA-thermograms of synthetized block copolymers: (a) P_51_D_47_, (b) P_122_D_54_, (c) P_122_D_96_, Figure S10: pH sensitive behavior for polymersomes encapsulating gold nanorod AuNR-761: (a) encapsulated with P_51_D_47_; (b) encapsulated with P_122_D_54_; (c) encapsulated with from P_122_D_96_.
