# Supplementary material for: Dual Responsive Polymersomes for Gold Nanorod and Doxorubicin Encapsulation: Nanomaterials with Potential Use as Smart Drug Delivery Systems
Source: Polymers (Basel). 2019 May 30;11(6):939. doi: 10.3390/polym11060939 (PMC6630455; doi:10.3390/polym11060939)
Supplement: Supplementary file 1 [file polymers-11-00939-s001.pdf]

SUPPLEMENTARY MATERIAL

FOR

Dual-responsive polymersomes for gold nanorod and doxorubicin encapsulation: Nanomaterials with potential use as smart drug delivery systems

Melissa DiazDuarte-Rodríguez[1], Norma A. Cortez-Lemus[1], Angel Licea-Claveríe[1]*, Jacob Licea-Rodriguez[2,3], Eugenio Méndez-Méndez[2].

1. Centro de Graduados e Investigación en Química. Tecnológico Nacional de México/Instituto Tecnológico de Tijuana, A.P. 1166. C.P. 22000 Tijuana, B. C., México.

2. Departamento de Física Aplicada. Centro de Investigación Científica y de Educación Superior de Ensenada, Carr. Ensenada-Tijuana No. 3918 Zona Playitas, A. P. 360, C.P. 22860 Ensenada, B. C., México.

3. Cátedras Conacyt, Centro de Investigación Científica y de Educación Superior de Ensenada.

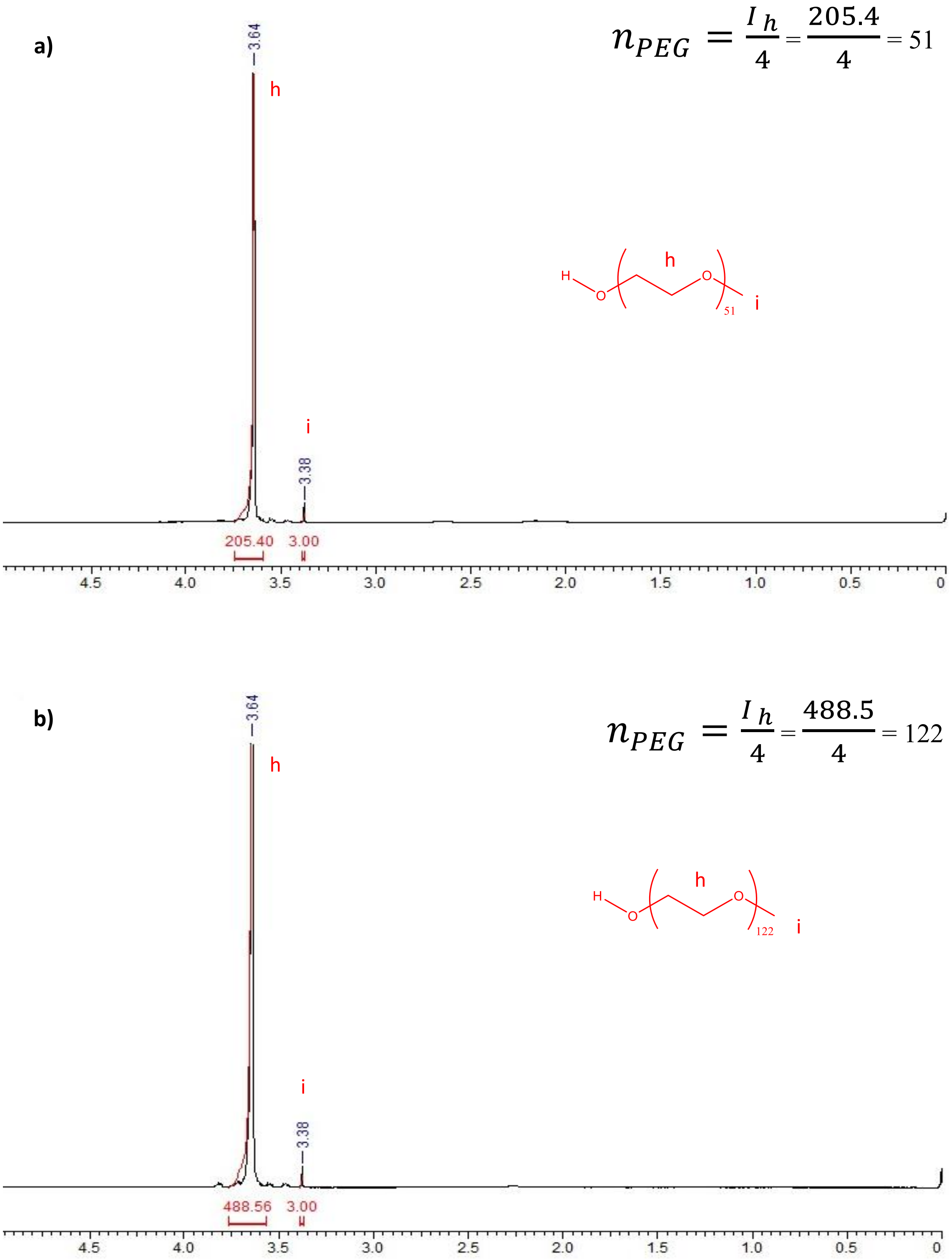

Figure S1. ^{1}H-NMR spectra for Sigma Aldrich® methoxy-Polyethyleneglycol of a) 2000 Da and b) 5000 Da.

The number of EG repetitive units was determined dividing the integration value of the signal *h* by the corresponding 4 Hydrogens.

Synthesis of the PEG_{122}-macroCTA

In a three necked flask, equipped with a magnetic stir bar, thermometer, and a Dean-Stark trap with a reflux condenser, methoxy-poly(ethylene glycol)5000 (M_n=5400 g/mol, 10.02 g, 1.86 mmol) was dissolved in toluene and refluxed for 48 h to eliminate water residues. Then, 4-cyano-4(dodecylsulfanylthiocarbonyl)sulfanyl pentanoic acid (1.6 g, 5.7 mmol), synthesized as described in the literature,[1] was dissolved in DCM (50 mL) and DMAP (0.24 g) was added to the three necked flask. The reaction mixture was cooled in an ice-bath and dicyclohexylcarbodiimide (0.82 g, 3.97 mmol) dissolved in DCM (6.0 mL) was added dropwise over 15 min. The reaction was allowed to proceed in an ice-cold bath for about 1 h, and then allowed to warm up to room temperature and stirred for 48 h. The solution was filtered to remove the precipitate. The solution was concentrated, and precipitated into ice-cold diethylether (x3). The yellow product was dried in vacuum oven at 25 ^oC for 24 h (43.2% yield).

^1H NMR (400 MHz, $CDCl_3$, δ ppm): 3.64 (CH_2CH_2O of the PEG chain), 3.38 (OCH_3, chain end of the PEG), 3.33 (SCH_2CH_2 of the CTA), 2.66-2.47 (CH_2CH_2OCO of the CTA), 2.51-2.16 (CH_2CH_2OCO), 1.87 (SCH_2CH_2 of the CTA) 1.70 (CH_3CN), 1.26 ($SCH_2(CH2)_9$-CH_3 of the CTA), 0.88 (CH3 of the CTA).

References

[1] G. Moad, Y.K. Chong, A. Postma, E. Rizzardo, S.H. Thang, Advances in RAFT polymerization: the synthesis of polymers with defined end-groups, Polymer, 46 (2005) 8458-8468. https://doi.org/10.1016/j.polymer.2004.12.061

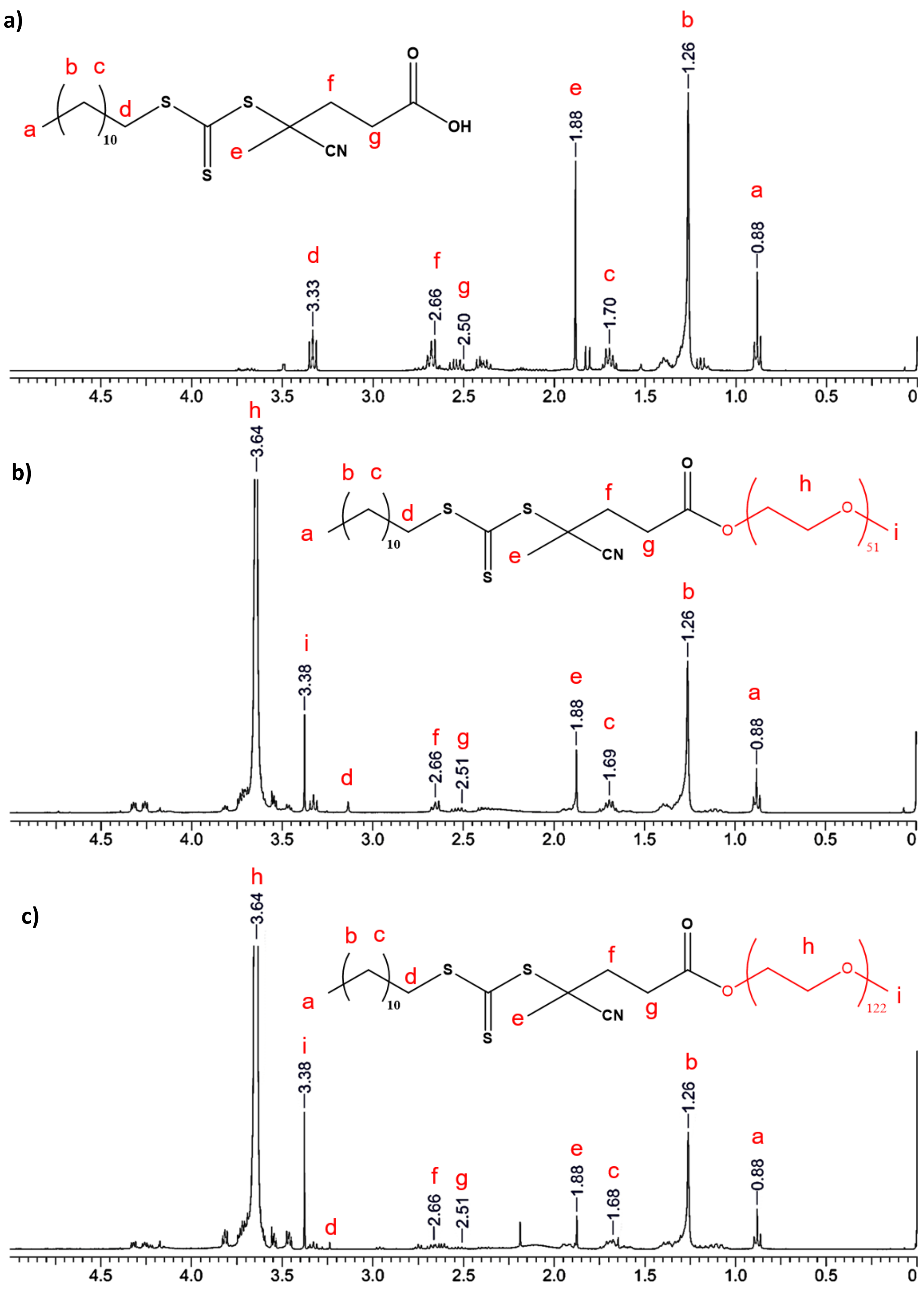

Figure S2. ^{1}H-NMR (400 MHz, $CDCl_3$) spectra for a) CTA: 4-cyano-4 (dodecylsulfanylthiocarbonyl) sulfanyl pentanoic acid, b) PEG_{51}-macroCTA, and c)PEG_{122}-macroCTA.

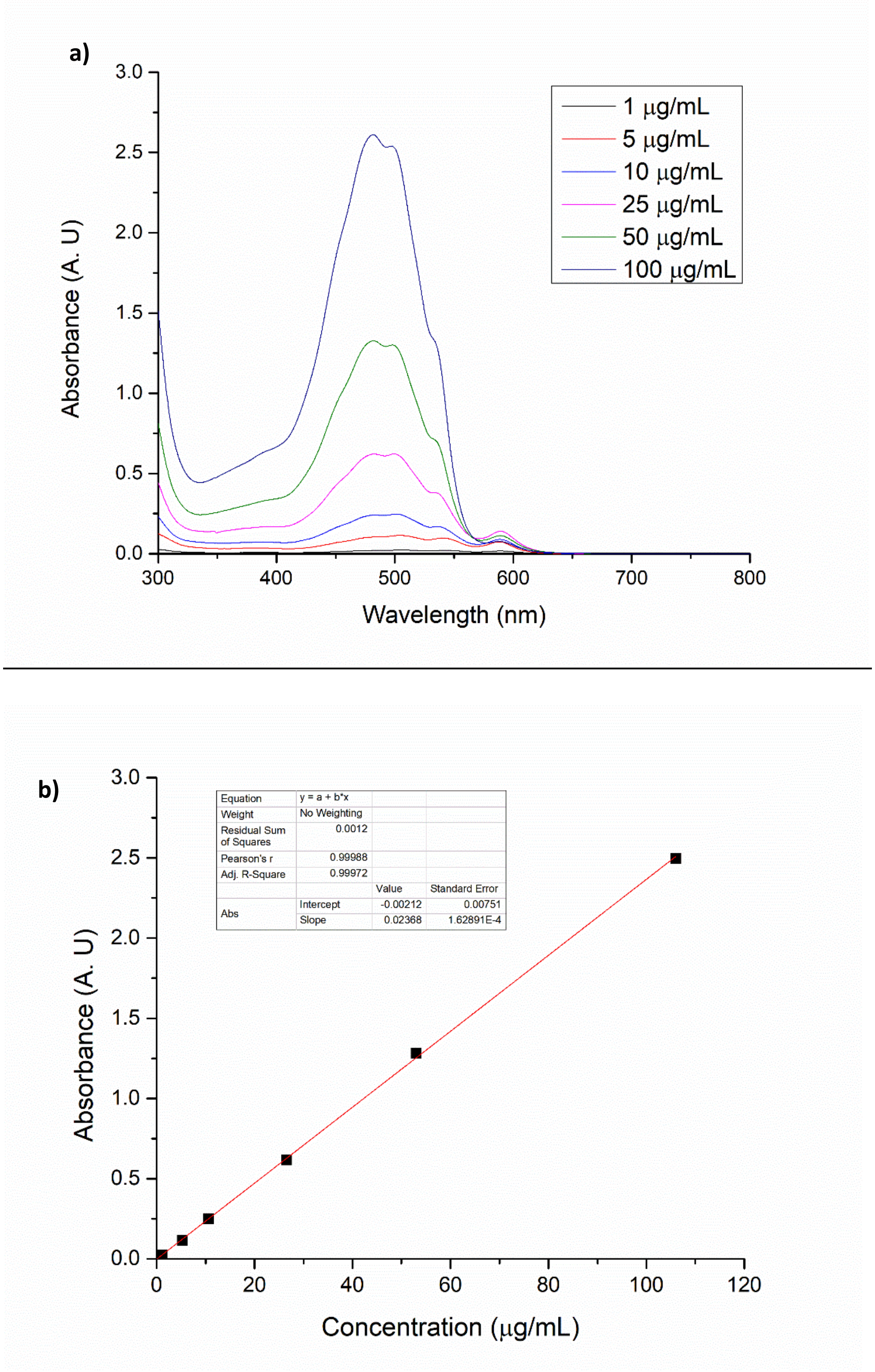

Figure S3. a)UV-Vis spectra of DOX in DMSO at different concentrations (0-100 μg/mL) and b) Calibration curve of DOX in DMSO at 484 nm.

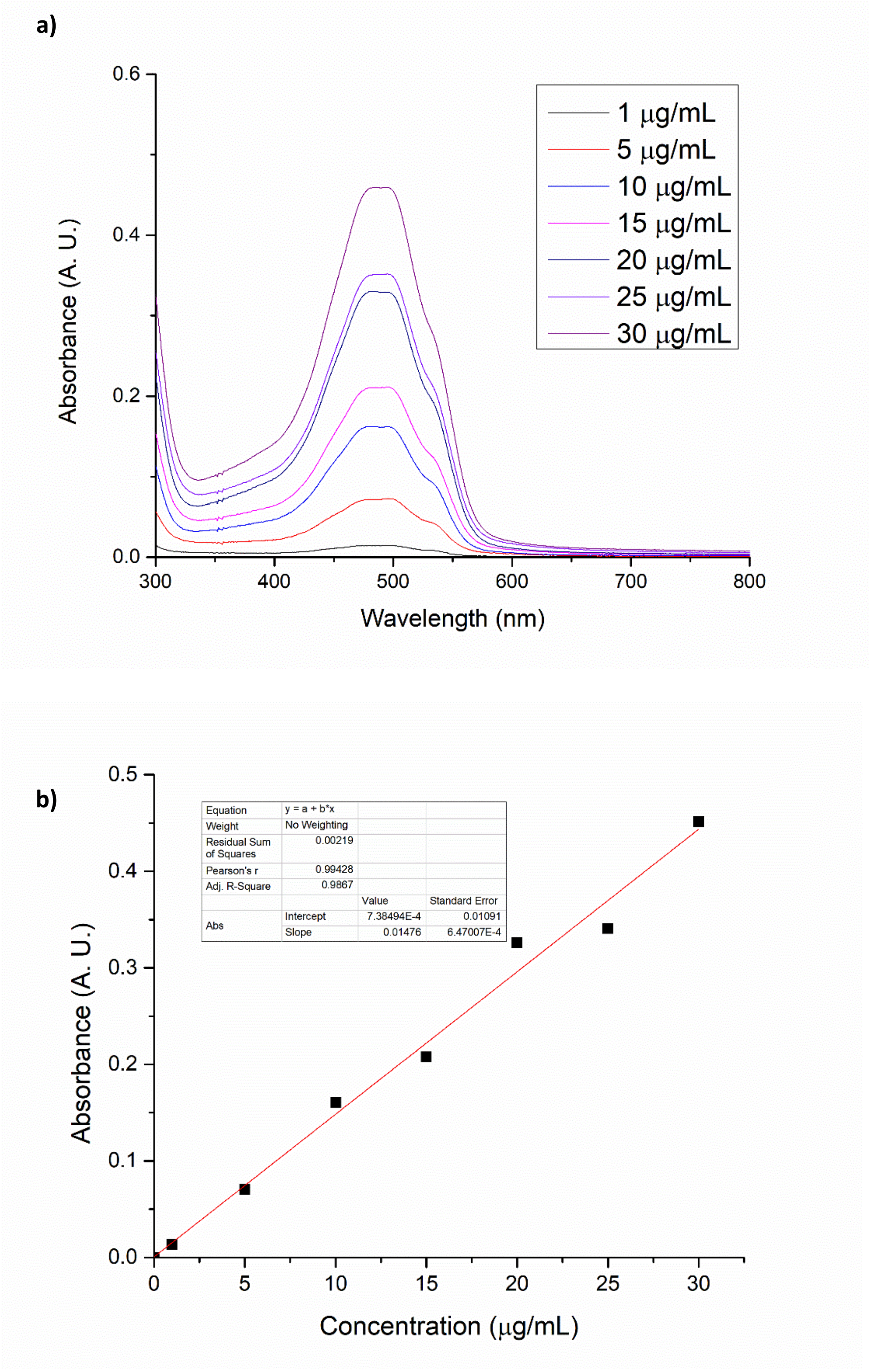

Figure S4. a)UV-Vis spectra of DOX in PBS at different concentrations (0-30 μg/mL) and b) Calibration curve of DOX in DMSO at 484 nm.

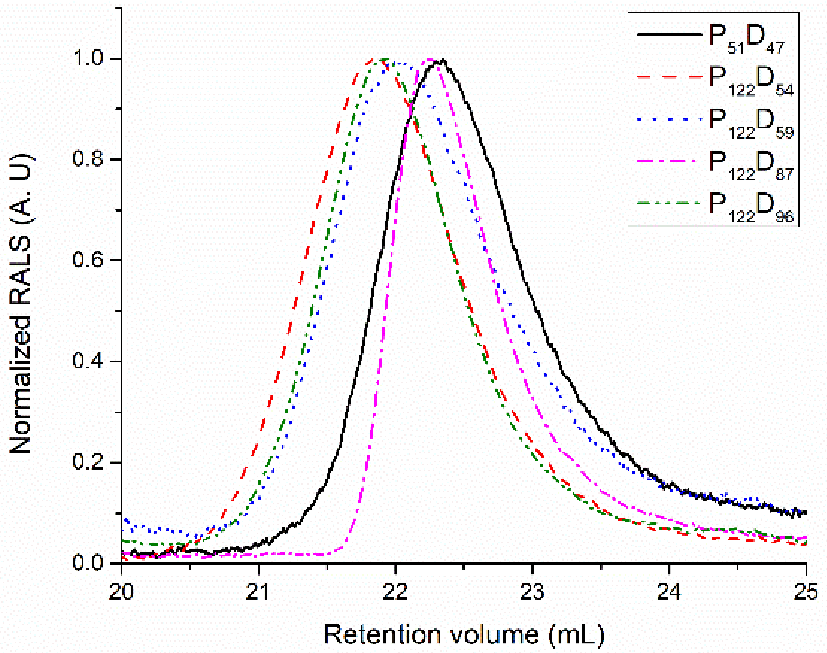

Figure S5. GPC traces (RALS-detector) of the copolymers $P_{51}D_{47}$ (PEG_{51}-*b*-$PDEAEM_{47}$), $P_{122}D_{54}$ (PEG_{122}-*b*-$PDEAEM_{54}$), $P_{122}D_{59}$ (PEG_{122}-*b*-$PDEAEM_{59}$), $P_{122}D_{87}$ (PEG_{122}-*b*-$PDEAEM_{87}$) and $P_{122}D_{96}$ (PEG_{122}-*b*-$PDEAEM_{96}$).

a)

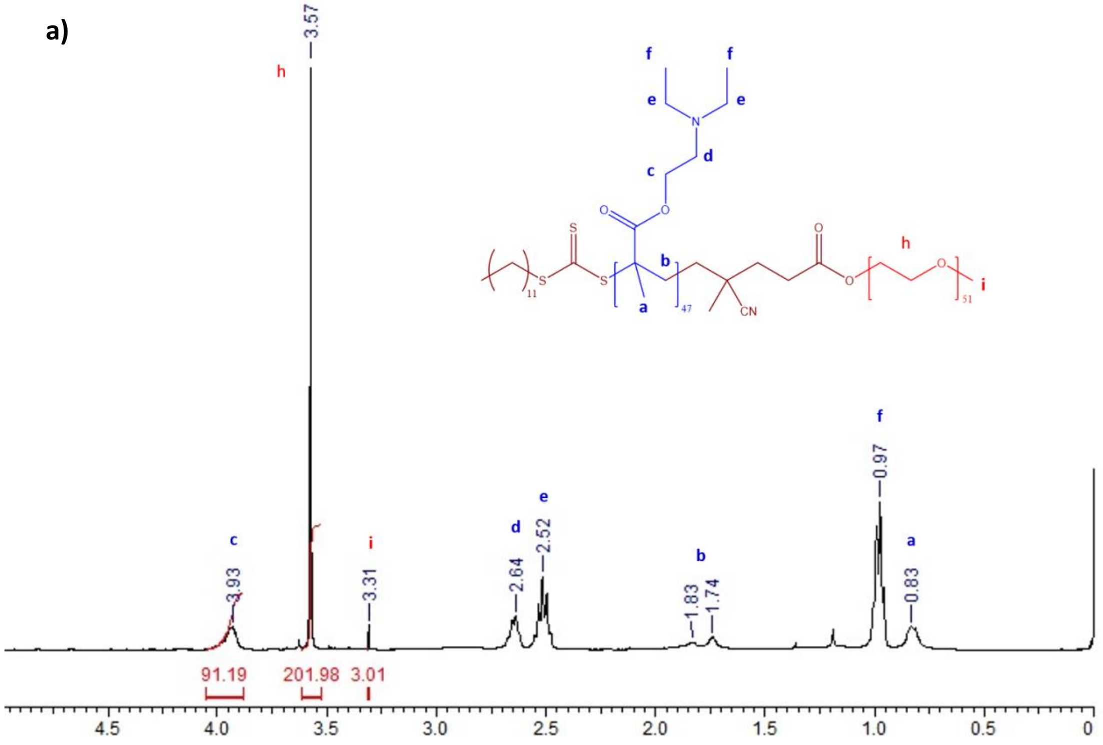
h
3.57
f
e
N
c
d
O
O
S
b
O
O
11
S
S
a
47
CN
O
h
O
51
i
c
3.93
i
3.31
d
2.64
e
2.52
b
1.83
1.74
f
0.97
a
0.83
91.19
201.98
3.01
4.5
4.0
3.5
3.0
2.5
2.0
1.5
1.0
0.5
0

b)

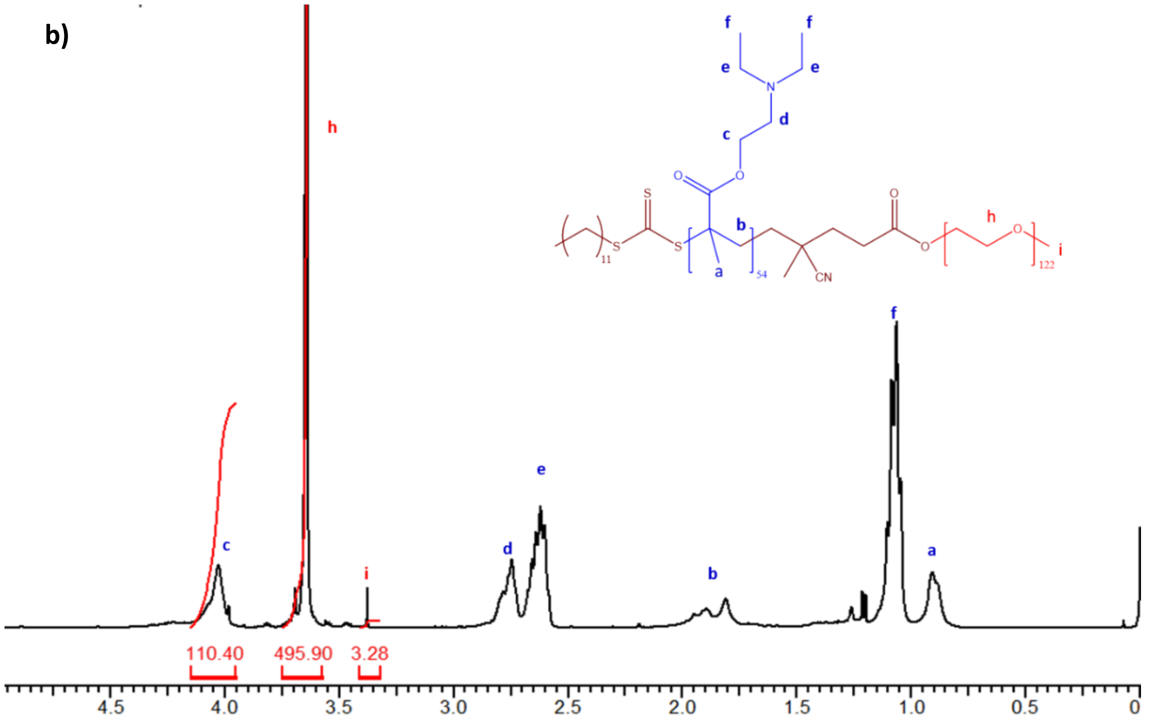
h
f
e
N
c
d
O
O
S
b
O
O
11
S
S
a
54
CN
O
h
O
122
i
c
i
d
e
b
f
a
110.40
495.90
3.28
4.5
4.0
3.5
3.0
2.5
2.0
1.5
1.0
0.5
0

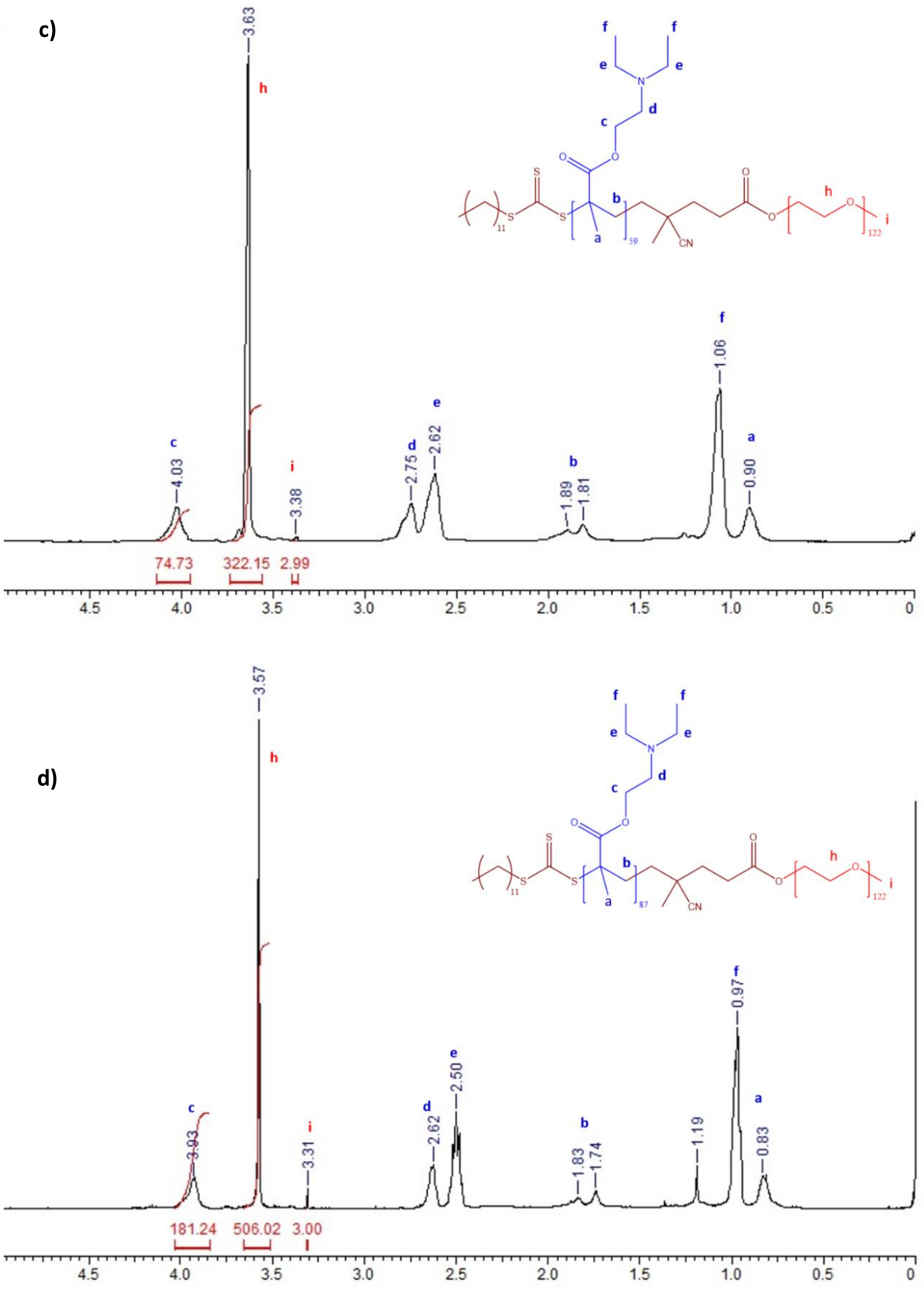

Figure S6. ^{1}H-NMR spectra (400 MHz, $CDCl_3$) of synthetized block copolymers: a)$P_{51}D_{47}$, b)$P_{122}D_{54}$, c)$P_{122}D_{59}$, d)$P_{122}D_{87}$

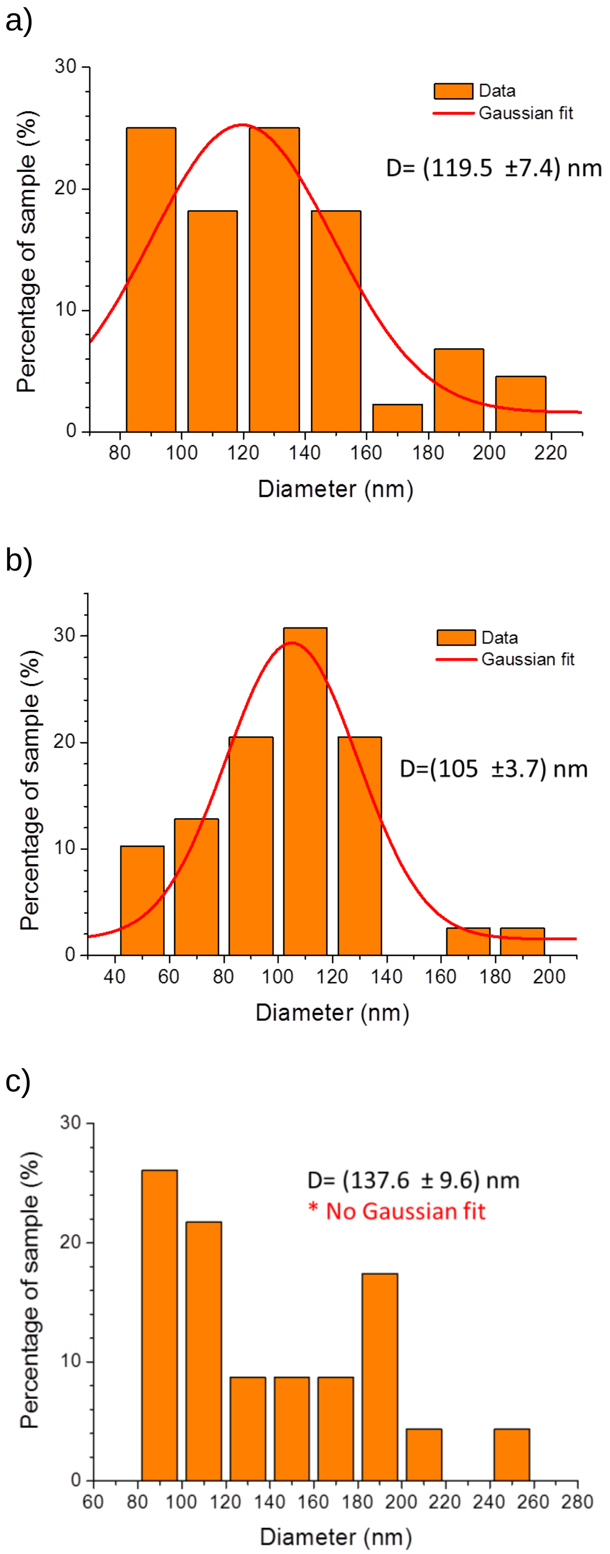

Figure S7. Statistics of TEM measurements of polymer aggregates: a)$P_{51}D_{47}$, b)$P_{122}D_{54}$, c)$P_{122}D_{96}$.

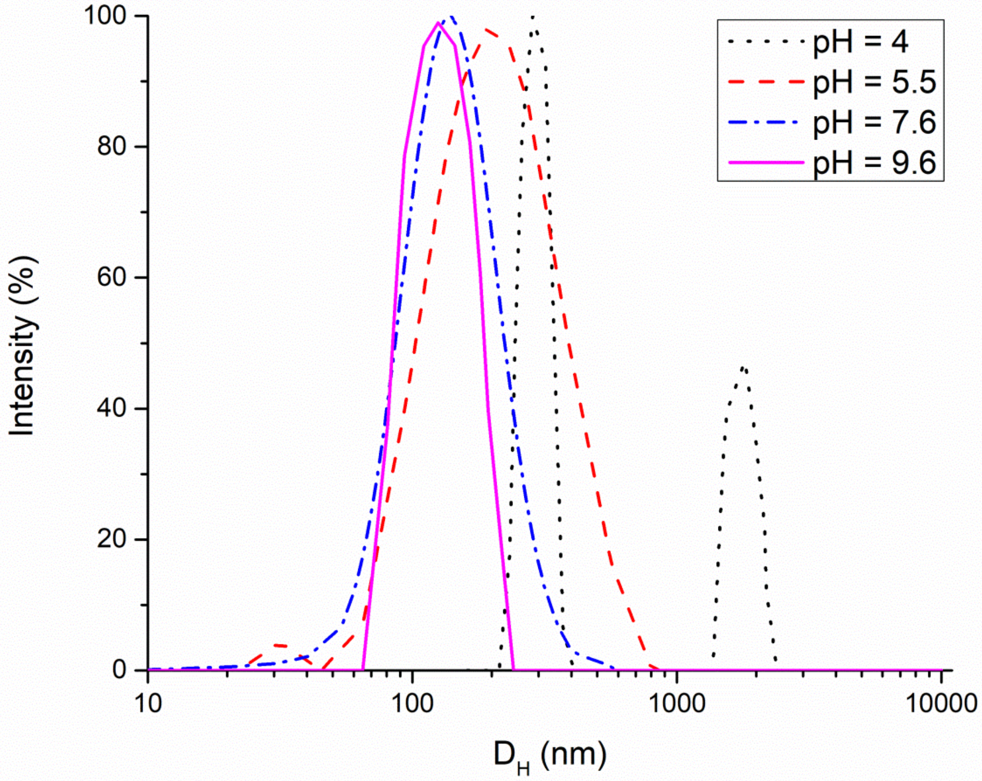

Figure S8. DLS distribution of sizes for the $P_{122}D_{96}$ polymer aggregates obtained by nanoprecipitation in water at different pH values.

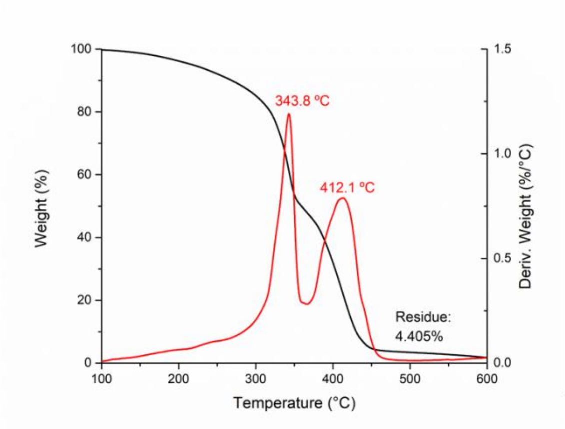

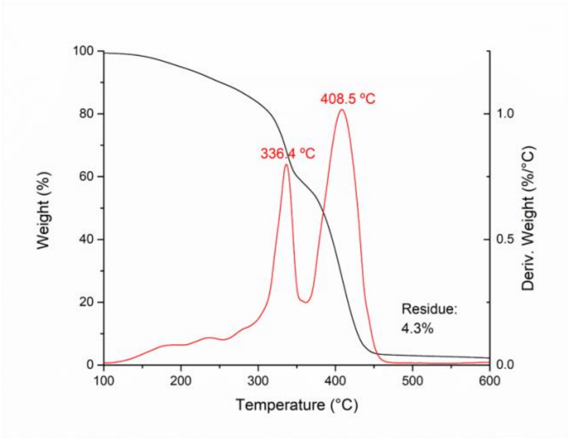

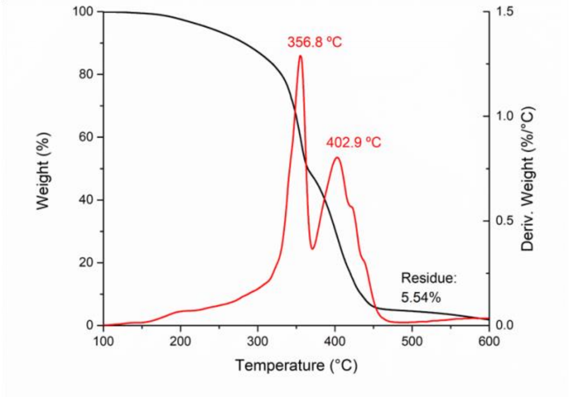

Polymer	T_{D1} (º C)	% D1	T_{D2} (º C)	% D2	Residue (%)
$P_{51}D_{47}$	343.8	48.2	412.1	47.4	4.4
$P_{122}D_{54}$	336.4	46.1	408.5	49.6	4.3
$P_{122}D_{96}$	356.8	50.9	402.9	43.6	5.5

Figure S9. TGA-thermograms of synthetized block copolymers: a)$P_{51}D_{47}$, b)$P_{122}D_{54}$, c) $P_{122}D_{96}$

a)

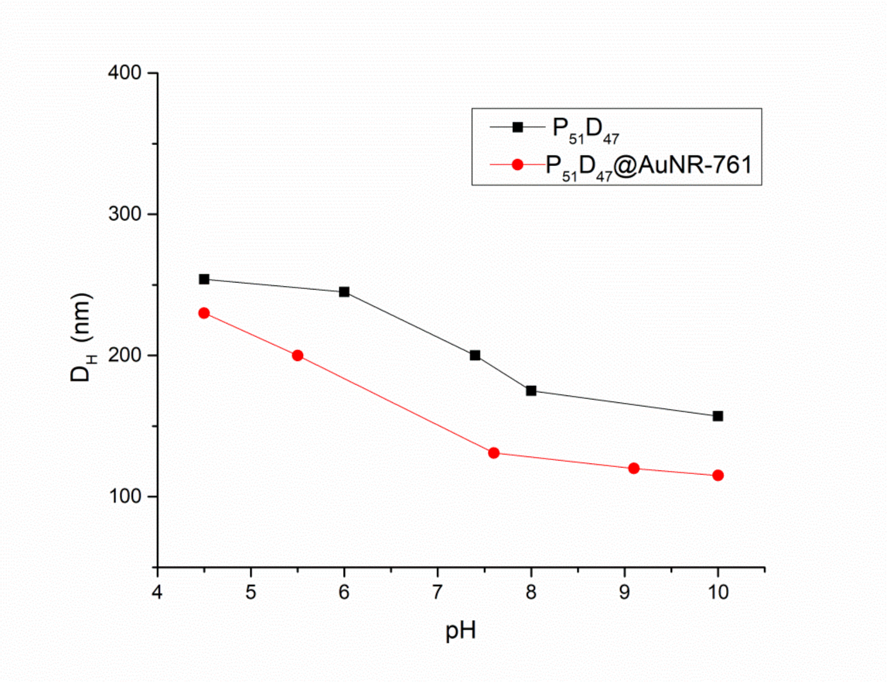

400
300
200
100
D_H (nm)
4
5
6
7
8
9
10
pH
$P_{51}D_{47}$
$P_{51}D_{47}$@AuNR-761

b)

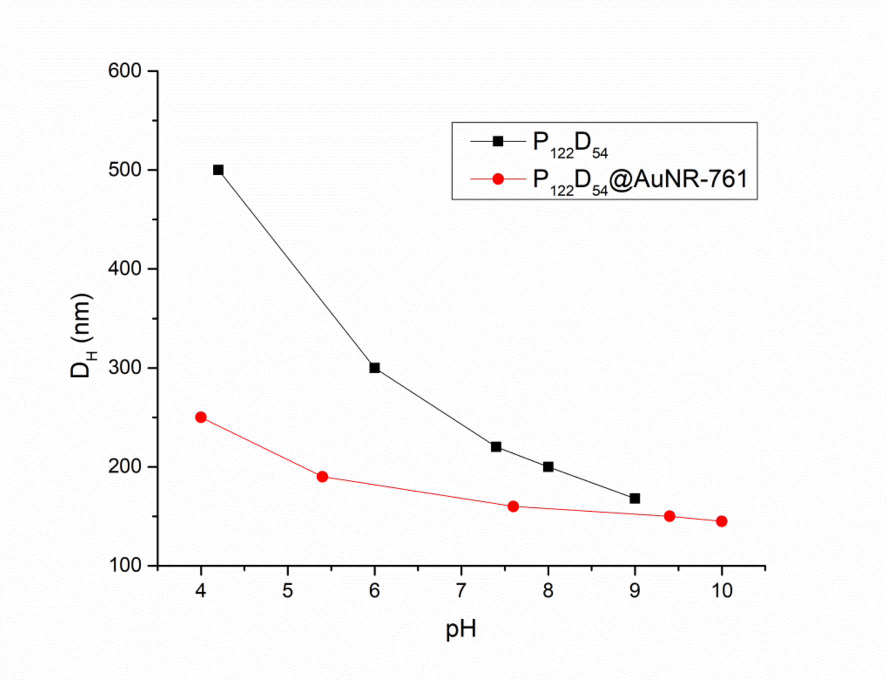

600
500
400
300
200
100
D_H (nm)
4
5
6
7
8
9
10
pH
$P_{122}D_{54}$
$P_{122}D_{54}$@AuNR-761

c)

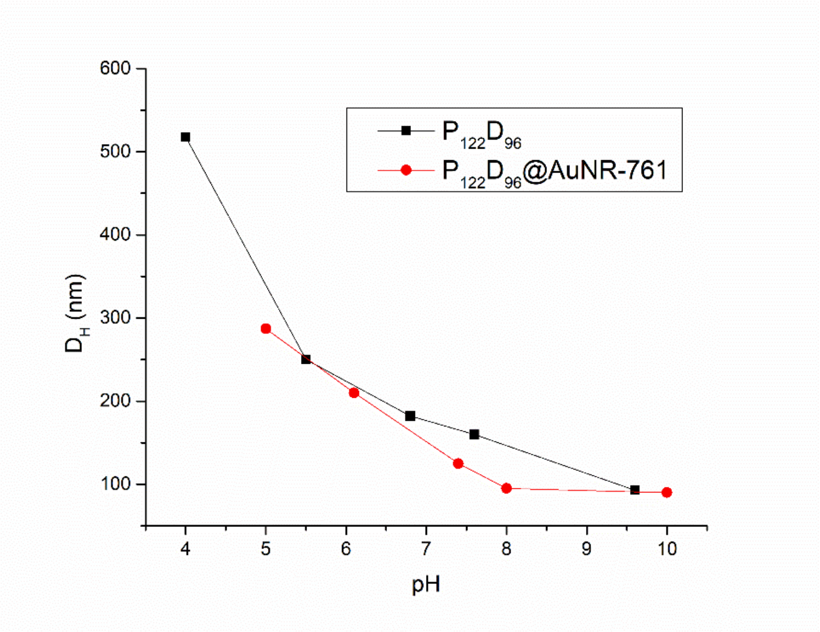

Figure S10. pH sensitive behavior for polymersomes encapsulating gold nanorod AuNR-761: a) encapsulated with $P_{51}D_{47}$; b) encapsulated with $P_{122}D_{54}$; c) encapsulated with from $P_{122}D_{96}$.
